# Statistical inference of entropy functions of generalized inverse exponential model under progressive type-II censoring test

**DOI:** 10.1371/journal.pone.0311129

**Published:** 2024-09-30

**Authors:** Qin Gong, Bin Yin

**Affiliations:** 1 College of Science, Jiangxi University of Science and Technology, Ganzhou, China; 2 Teaching Department of Basic Subjects, Jiangxi University of Science and Technology, Nanchang, China; Federal University of Pernambuco: Universidade Federal de Pernambuco, BRAZIL

## Abstract

This article explores the estimation of Shannon entropy and Rényi entropy based on the generalized inverse exponential distribution under the condition of stepwise Type II truncated samples. Firstly, we analyze the maximum likelihood estimation and interval estimation of Shannon entropy and Rényi entropy for the generalized inverse exponential distribution. In this process, we use the bootstrap method to construct confidence intervals for Shannon entropy and Rényi entropy. Next, we select the gamma distribution as the prior distribution and apply the Lindley approximation algorithm to calculate `estimates of Shannon entropy and Rényi entropy under different loss functions including Linex loss function, entropy loss function, and DeGroot loss function respectively. Afterwards, simulation is used to calculate estimates and corresponding mean square errors of Shannon entropy and Rényi entropy in GIED model. The research results show that under DeGroot loss function, estimation accuracy of Shannon entropy and Rényi entropy for generalized inverse exponential distribution is relatively high, overall Bayesian estimation performs better than maximum likelihood estimation. Finally, we demonstrate effectiveness of our estimation method in practical applications using a set of real data.

## 1. Introduction

With the continuous progress of technology, consumers have increasingly higher requirements for product quality. In order to meet market demand, we need to conduct reliability and life tests to evaluate the performance and durability of the product. For instance, Zhang et al. [1] conducted a reliability analysis on the copula-based partially accelerated competition risk model. Alotaibi et al. [2] analyzed the constant stress accelerated life test of XLindley distribution. However, when conducting these experiments, we often face various limitations such as time, cost, and experimental conditions. These limitations prevent us from fully observing the complete lifespan of all products, and there may be some products that fail before or during testing, making it impossible to continue with life testing. To more accurately estimate the lifetime of products for reliability assessment, decision-making, or product improvement purposes, censoring samples are used in lifetime testing. Considering factors such as long product lifetimes and high testing costs, it is necessary to adopt more efficient and cost-effective testing methods. Therefore, the concept of progressive censoring sampling is introduced to further improve the accuracy of reliability and lifetime distribution estimation for products. Compared to traditional censoring methods, the progressive censoring method demonstrates enhanced flexibility in product life testing because it gradually adjusts the censorship samples to better align with the distribution characteristics of product lifespan. This approach improves both testing efficiency and accuracy by reducing data loss caused by premature sample removal and retaining a larger number of longer-lived samples, thus enhancing data utilization and obtaining more precise lifetime estimates. Additionally, the progressive censoring method allows for reduced testing time, smaller sample sizes, lower testing costs while enabling more reliable risk assessment based on accurate lifespan estimation. As a result, it provides a more scientific basis for product design and decision-making while comprehensively evaluating long-term performance. In recent research, significant progress has been made in the field of statistical analysis of progressive censoring experiments. For instance, Chakraborty et al. [3] conducted a comprehensive analysis of the joint cumulative entropy under progressive Type-II censoring (PC-II) samples. Alsadat et al. [4] investigated the parameter estimation problem for unit semi-logical geometric distribution under progressive Type-II right censoring samples. Lone et al. [5] studied a stress strength reliability model based on a balanced joint progressive censoring scheme and performed parameter estimation and reliability analysis on Burr XII type distribution samples using classical and Bayesian methods, confirming the effectiveness of the adopted approach. Additionally, Alsadat et al. [6] analyzed the properties of Kumaraswamy’s modified inverse Weibull distribution in samples with progressive first failure censoring. Berred and Stepanov [7] examined the distribution characteristics and asymptotic behavior of exponential intervals under PC-II samples. Moreover, Alotaibi et al. [8] conducted research on Frechet distribution prediction based on review data in fields such as medicine and technical sciences. These research findings demonstrate continuous advancements and enhancements in statistical analysis methods for progressive censoring experiments. Progressive censoring testing can be divided into progressive Type-I censoring testing and PC-II testing, with a focus on PC-II testing in this paper. Based on the concept proposed by Alotaibi [8], PC-II testing can be described as follows:

If there are *n* products undergoing lifetime testing, and the time of observing the first failed product is recorded as *X*_1:*m*:*n*_, *R*_1_ products need to be excluded out of the remaining *n*−1 products that have not failed. For the second failed product, the same procedure should be replicated, observed as *X*_2:*m*:*n*_, by removing *R*_2_ products from the remaining *n*−2−*R*_1_ non-failed products, by extension, the time at which the occurrence of the m-th faulty product is observed is recorded as *X*_*m*:*m*:*n*_. At this stage, the experiment concludes, and any remaining *n*−*m*−*R*_1_−*R*_2_−⋯−*R*_*m*_ products are automatically removed.

Abouammoh and Alshingiti [9] proposed the generalized inverse exponential distribution (GIED), a novel distribution type, which combines the generalized exponential distribution (GED) with the inverse exponential distribution (IED) and is an extended form of the IED. This distribution is widely used in survival analysis, reliability engineering, and communication fields [10]. Compared with the exponential distribution and IED, GIED introduces additional parameters, making it more flexible and able to better adapt to various data situations and perform fitting. Therefore, in recent years, the statistical properties of this distribution have been extensively examined by numerous scholars. For example, Bakoban and Aldahlan [11] studied Bayesian estimation of shape parameters for GIED under complete samples. Hassan et al. [12] assumed that strength and stress follow GIED with different shape parameters as random variables under ranked set sampling (RSS) and simple random sampling (SRS), and analyzed the reliability estimation of stress intensity. Liu and Xi [13] studied the Bayesian estimation of GIED parameters under timed censoring samples. Below we provide the definition of GIED:

Let *X* denote a stochastic variable which follows the GIED. The probability density function (PDF) and cumulative distribution function (CDF) of this distribution are given by:

$$f(x;\lambda ,\beta )=\frac{\lambda \beta }{x^{2}}e^{-\frac{\lambda }{x}}{\left(1-e^{-\frac{\lambda }{x}}\right)}^{\beta -1},\;x>0,\lambda ,\beta >0,$$ (1)

$$F(x;\lambda ,\beta )=1-{\left(1-e^{-\frac{\lambda }{x}}\right)}^{\beta },\;x>0,\lambda ,\beta >0.$$ (2)

Here *β* is the shape parameter, and *λ* is the scale parameter.

**Fig 1:**
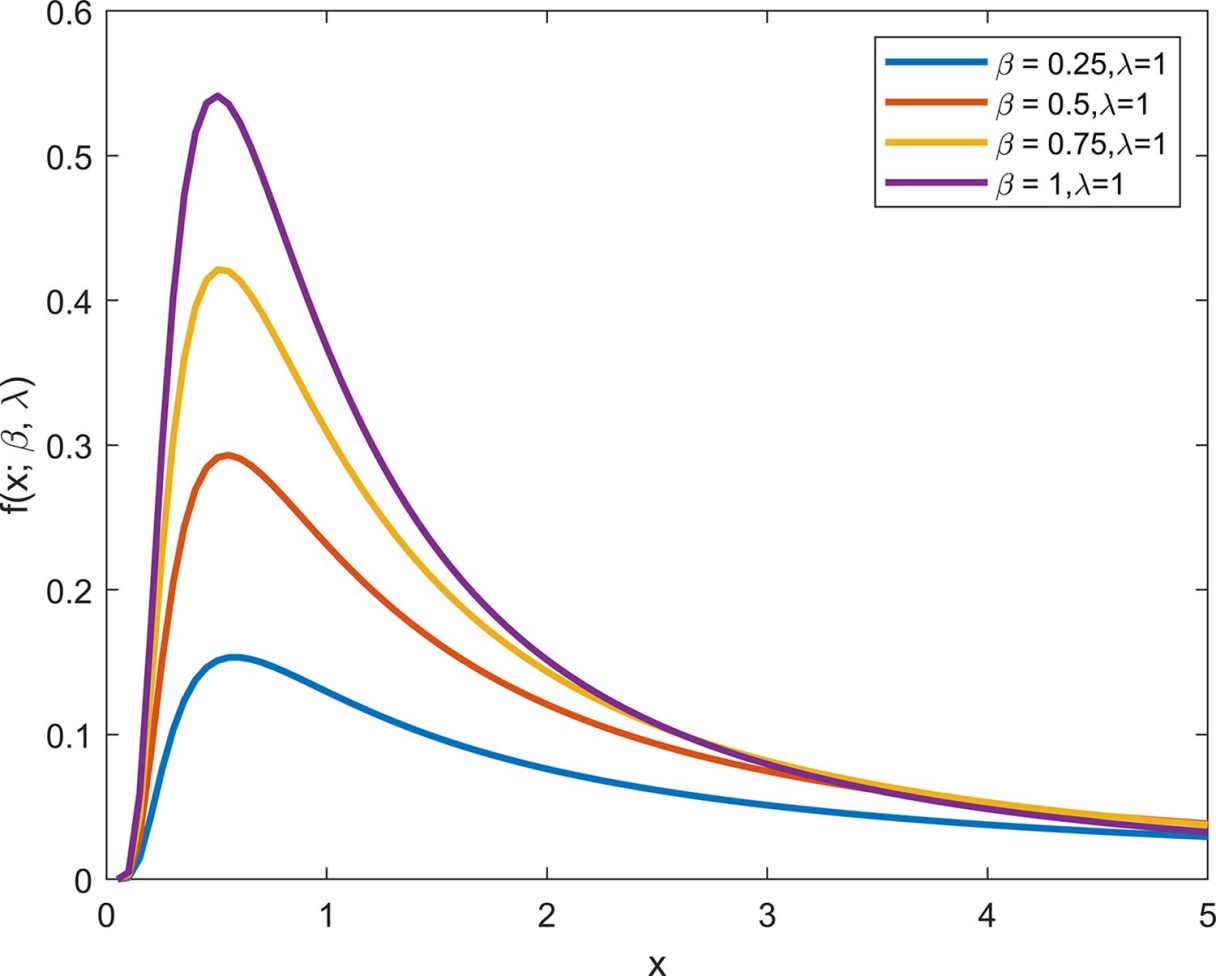
PDF plots of GIED.

**Fig 2:**
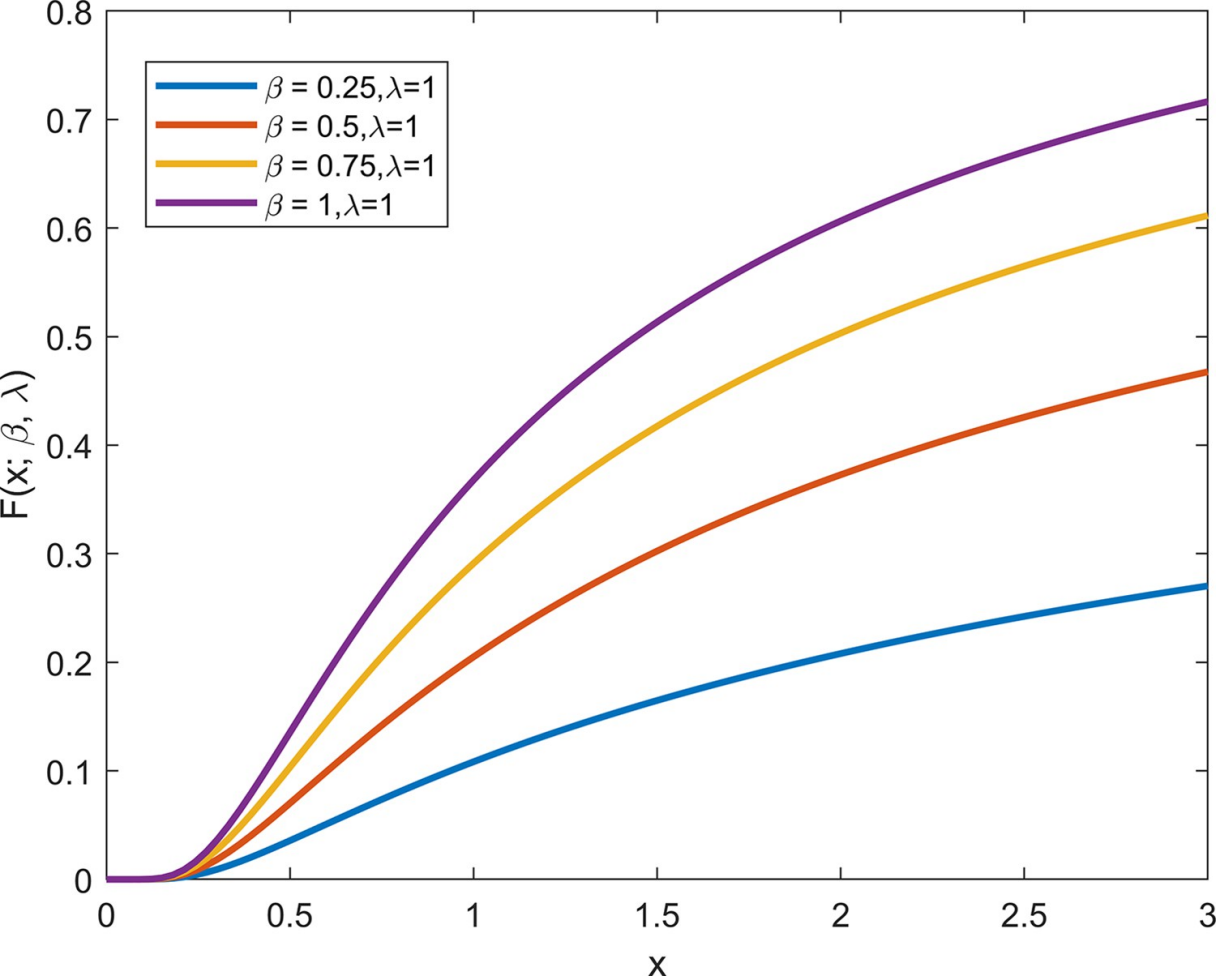
CDF plots of GIED.

From Figs 1 and 2, it can be observed that when *λ* is fixed, the PDF and CDF exhibit different trends with varying *β*. When *β* = 1, the GIED is IED.

Although GIED, as an emerging distribution, has shown great potential in fields such as survival analysis and reliability engineering and has attracted the attention of many scholars, research on statistical inference of GIED in PC-II samples is still relatively scarce. In view of this, this article aims to address the shortcomings in this area and promote the application of GIED in censoring data scenarios. This article combines PC-II samples with the GIED model to achieve accurate estimation of GIED parameters through parameter estimation and hypothesis testing while verifying the applicability of the model. Entropy, an important concept in information theory, also plays a significant role in practical applications. It can be used to evaluate the uncertainty and risk level of random variables, thereby assisting in risk assessment and decision-making [14]. Additionally, entropy value can serve as a criterion for selecting distribution models where higher entropy values indicate stronger uncertainty and randomness within data. Based on this understanding, appropriate adjustments are made to model selection criteria. This article delves into estimating entropy within the GIED model while further validating its applicability through parameter estimation and hypothesis testing.

This study is based on PC-II samples and primarily explores the estimation of entropy in GIED. In Section 1, the basic concepts and properties of PC-II experiments and the GIED model are introduced. In Section 2, the terms for Shannon entropy and Rényi entropy in GIED are deduced and proved. In Section 3, we derived maximum likelihood (ML) estimates for Shannon entropy and Rényi entropy, and confidence intervals (CIs) for Shannon entropy and Rényi entropy are constructed using the bootstrap method. Section 4 uses a gamma distribution as a prior, and Bayesian inference for the estimation of Shannon entropy and Rényi entropy under the Linex loss function (LLF), entropy loss function (ELF), and DeGroot loss function (DLF) is performed. The Lindley approximation algorithm is applied to compute the Bayesian estimator (BE). In Section 5, numerical results are obtained through Monte Carlo simulations to validate the effectiveness of the proposed estimation methods. In Section 6, the constructed model is applied to real data, and the validity of the employed estimation approaches is verified. Finally, in Section 7, a thorough discussion of the research results is provided, and corresponding conclusions are drawn.

## 2. Entropy under GIED

Shannon entropy, introduced by Shannon [15], it is a fundamental concept in information theory that serves to gauge the degree of uncertainty or stochasticity in information. The definition of Shannon entropy is given below:

$$H_{S}(f)=-\int_{-\infty }^{+\infty }f(x)\ln f(x)dx,$$ (3)

where *f*(*x*) represents the PDF of the stochastic variable *X* that is continuous. With the development of information theory, the concept of Shannon entropy has been widely applied in various fields such as communication, data mining and machine learning, and statistical physics. Flores-Gallegos and Flores-Gómez [16] revealed the relationship between Shannon entropy and chemical hardness and applied the derived equations to molecular ensembles. Joshi [17] studied the variations in Shannon entropy under changes in constraint potential parameters and Debye screening parameters. Flores-Gallegos [18] analyzed the trends in the first derivative of Shannon entropy with respect to electron number and spin density.

Rényi entropy is an extended form of information entropy proposed by Rényi in 1960 [19]. Unlike Shannon entropy, Rényi entropy introduces a parameter *α*, which can adjust the properties of entropy to some extent. The definition of Rényi entropy is given below:

$$H_{R}(f)=\frac{1}{1-\alpha }\ln\int_{-\infty }^{+\infty }{\left[f(x)\right]}^{\alpha }dx,\alpha (\neq 1)>0,$$ (4)

where *f*(*x*) represents the PDF of the stochastic variable *X* that is continuous. Rényi entropy is an extension of entropy in information theory and plays an important role in various fields such as information theory, statistics, and complex networks. Significant progress has been made in the study of Rényi entropy in existing literature. Chennaf and Ben Amor [20] analyze the mathematical properties of Rényi entropy and partial Rényi entropy, applying them to measure the uncertainty of uncertain random variables. They also apply partial Rényi entropy to optimize the selection of uncertain random returns in finance. Tian and Xu [21] discussed the problem of calculating Rényi entropy in AdS (3)/(B) CFT2. Kayid and Shrahili [22] used the signature of the system to determine the Rényi entropy of the past longevity of a interrelated system, in order to evaluate its predictability.

**Theorem 1.** *Let X denote a stochastic variable which follows the GIED*. *The Shannon entropy of GIED is given by*:

$$H_{S}=-\ln\lambda \beta +2\beta \sum_{i=0}^{\infty }\left(\begin{array}{c} \beta -1\\ i \end{array}\right){\left(-1\right)}^{i}\frac{\Gamma (1)\ln(1+i)-\Gamma^{\prime }(1)}{1+i}+\psi (\beta +1)-\psi (1)+\frac{\beta -1}{\beta },$$ (5)

*where* Γ(.) *denotes the gamma function*, *and ψ*(.) *denotes the digamma function*.

**Proof.** See S1 Appendix.

**Theorem 2.** *Let X denote a stochastic variable which follows the GIED*. *The Rényi entropy of GIED is given by*:

$$H_{R}=\frac{1}{1-\alpha }\ln[\lambda^{-\alpha +1}\beta^{\alpha }\sum_{i=0}^{\infty }\left(\begin{array}{c} \alpha \beta -\alpha \\ i \end{array}\right){\left(-1\right)}^{i}\frac{\Gamma (2\alpha -1)}{{\left(\alpha +i\right)}^{2\alpha -1}}],$$ (6)

*where* Γ(.) *denotes the gamma function*.

**Proof.** See S2 Appendix.

## 3. ML estimation

Suppose $X_{1:m:n},X_{2:m:n},\cdots ,X_{m:m:n}$ represents m PC-II samples observed out of n test samples in total, following the GIED defined by Eq (1), according to Abo-Kasem [23], the likelihood function (LF) is:

$$L(\beta ,\lambda |\underline{x})=\xi \prod_{i=1}^{m}f(x_{i:m:n}){\left[1-F(x_{i:m:n})\right]}^{R_{i}}=\xi {\left(\lambda \beta \right)}^{m}\prod_{i=1}^{m}x_{i}^{-2}e^{-\frac{\lambda }{x_{i}}}{\left(1-e^{-\frac{\lambda }{x_{i}}}\right)}^{\beta R_{i}+\beta -1},$$ (7)

where $\xi =n(n-R_{1}-1)(n-R_{1}-R_{2}-2)\cdots (n-R_{1}-R_{2}-\cdots R_{m-1}-m+1)$, *X*_*i*:*m*:*n*_ represents the observed values of the *X*_*i*:*m*:*n*_ samples, $\underline{x}=(x_{1:m:n},x_{2:m:n},\cdots ,x_{m:m:n})$.

According to Eq (7), the logarithm of the LF is obtained as:

$$l(\beta ,\lambda |\underline{x})=\ln L(\beta ,\lambda |\underline{x})=\ln\xi +m\ln\lambda +m\ln\beta -2\sum_{i=1}^{m}\ln x_{i}-\lambda \sum_{i=1}^{m}\frac{1}{x_{i}}+\sum_{i=1}^{m}\left(\beta R_{i}+\beta -1)\ln(1-e^{-\frac{\lambda }{x_{i}}}\right).$$

Therefore, Eqs (8) and (9) are obtained:

$$\frac{\partial l(\beta ,\lambda )}{\partial \beta }=\frac{m}{\beta }+\sum_{i=1}^{m}\left(R_{i}+1)\ln(1-e^{-\frac{\lambda }{x_{i}}}\right),$$ (8)

$$\frac{\partial l(\beta ,\lambda )}{\partial \lambda }=\frac{m}{\lambda }-\sum_{i=1}^{m}\frac{1}{x_{i}}+\sum_{i=1}^{m}(\beta R_{i}+\beta -1)\frac{e^{-\frac{\lambda }{x_{i}}}}{x_{i}(1-e^{-\frac{\lambda }{x_{i}}})}.$$ (9)

**Fig 3:**
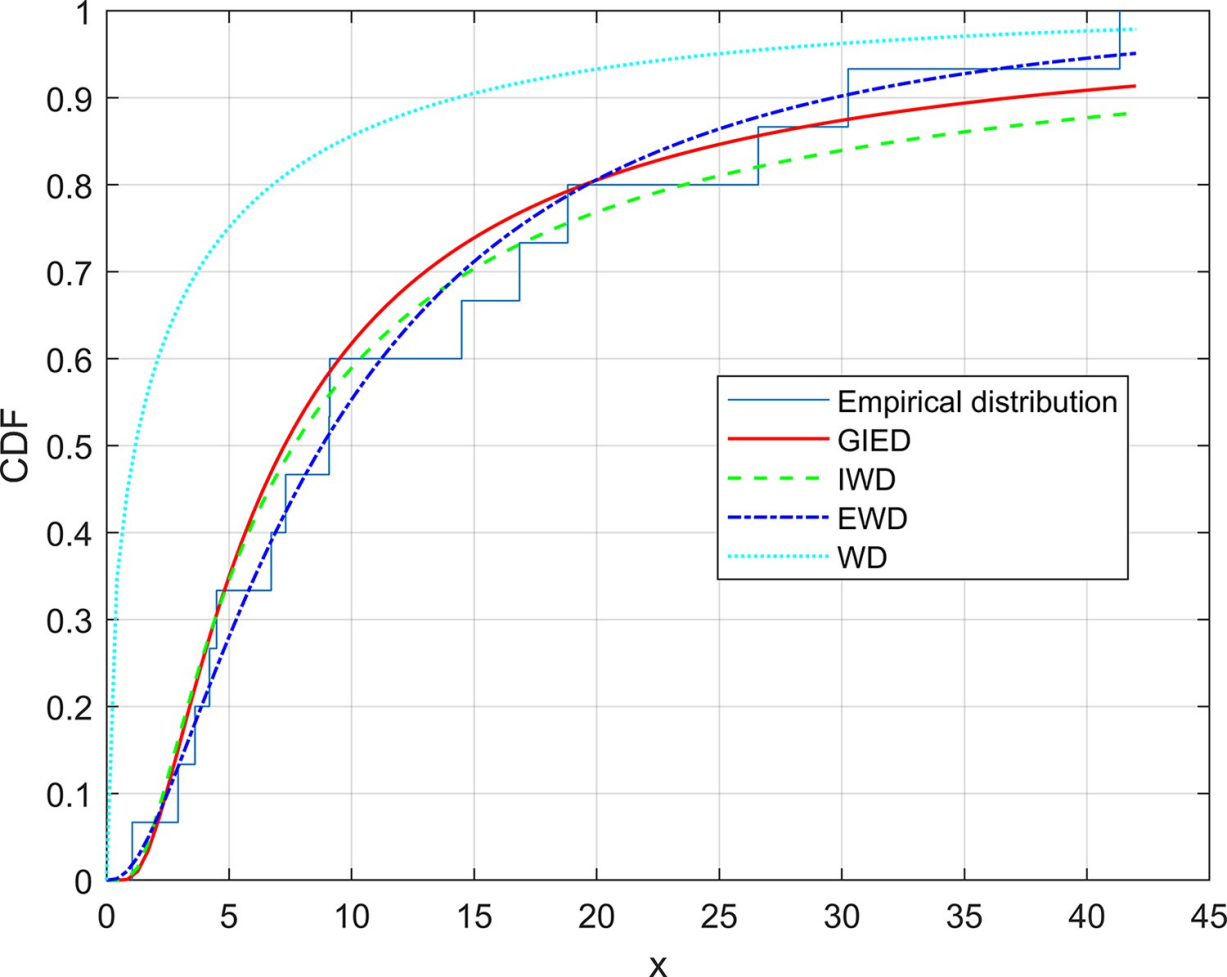
Experience distribution diagram of patient survival time and distribution function diagrams of various models function plot of GIED.

By setting the right-hand side of Eqs (8) and (9) to zero and solving these two equations, we obtain the roots, which are the ML estimates of *β* and *λ*. Moreover, the solutions obtained from Eqs (8) and (9) exist and are unique. However, proving directly that solutions to the nonlinear Eqs (8) and (9) exist and are unique can prove challenging. Therefore, we can provide evidence through visualizing the logarithm likelihood equation, as depicted in the real data analysis presented in Section 6 of Fig 3. Since we cannot obtain an analytical solution directly for Eqs (8) and (9), we can consider using numerical methods to obtain the ML estimates of *β* and *λ*. In the following, we will introduce the algorithm steps of the dichotomy method.

Step 1: Given an error capacity *ε*, determine the interval range [*λ*_*L*_, *λ*_*U*_] such that *f*(*λ*_*L*_)⋅*f*(*λ*_*U*_)<0.

Step 2: Find the point *λ*_*M*_ in the middle of the range [*λ*_*L*_, *λ*_*U*_] and substitute it into *f*(*λ*) to calculate *f*(*λ*_*M*_).

Step 3: If *f*(*λ*_*M*_) = 0, then $\hat{\lambda }=\lambda_{M}$, the algorithm ends; If *f*(*λ*_*L*_)⋅*f*(*λ*_*M*_)<0, then *λ*_*U*_ = *λ*_*M*_; If *f*(*λ*_*U*_)⋅*f*(*λ*_*M*_)<0, then *λ*_*L*_ = *λ*_*M*_.

Step 4: Repeat step 3 until |*λ*_*L*_−*λ*_*U*_|<*ε*, and the algorithm ends.

Because of the invariance property of ML estimation, we substitute the parameter estimates $\hat{\beta }$ and $\hat{\lambda }$ obtained from the dichotomy method into Eqs (5) and (6), we get:

$${\hat{H}}_{S}=-\ln\hat{\lambda }\hat{\beta }+2\hat{\beta }\sum_{i=0}^{\infty }\left(\begin{array}{c} \hat{\beta }-1\\ i \end{array}\right){\left(-1\right)}^{i}\frac{\Gamma (1)\ln(1+i)-\Gamma^{\prime }(1)}{1+i}+\psi (\hat{\beta }+1)-\psi (1)+\frac{\hat{\beta }-1}{\hat{\beta }},$$ (10)

$${\hat{H}}_{R}=\frac{1}{1-\alpha }\ln[{\hat{\lambda }}^{-\alpha +1}{\hat{\beta }}^{\alpha }\sum_{i=0}^{\infty }\left(\begin{array}{c} \alpha \hat{\beta }-\alpha \\ i \end{array}\right){\left(-1\right)}^{i}\frac{\Gamma (2\alpha -1)}{{\left(\alpha +i\right)}^{2\alpha -1}}].$$ (11)

In statistics, CIs are used to describe the range of uncertainty in parameter estimation results, representing the possible range of true values of parameters at a given confidence level. There are various methods for constructing CIs, and choosing the appropriate method mainly depends on the estimated parameter type and sample distribution. The bootstrap method is a resampling technique in statistics used to estimate the sampling distribution of statistics, construct CIs, and perform hypothesis testing. It is generated by repeatedly sampling with replacement from the original sample to simulate multiple virtual sample sets, thereby avoiding assumptions about the population distribution. By conducting repeated sampling and parameter estimation on a virtual sample set, the sampling distribution of parameter estimation can be obtained, and then CIs can be constructed. Abundant research has been conducted on the bootstrap method in the existing literature. Kanwal and Abbas [24] investigated parameter estimation of the Frechet distributed process capability index and established a corresponding bootstrap CI. Li et al. [25] proposed a novel bootstrap method for small sample hydrologic frequency analysis, demonstrating its superior accuracy compared to traditional methods, particularly with limited sample sizes. Hwang et al. [26] effectively addressed uncertainty in landslide probability analysis caused by insufficient data using the bootstrap method and combined it with point estimation to propose a new approach. Su et al. [27] introduced an improved bootstrap method for estimating fatigue properties of materials and components under small sample sizes. Dudorova et al. [28] employed a bootstrap analysis method to study preferences of Egyptian fruit bat pups, suggesting that these preferences be transcribed based on behavioral test data presented therein. These studies provide valuable application cases and theoretical foundations for diverse fields and applications of the bootstrap method. Maiti et al. [29] estimated the Shannon entropy and Rényi entropy of GED using progressive censoring data, and constructed CIs for entropy using bootstrap method.

In this section, we utilize the bootstrap-t (Boot-t) method to construct CIs for the GIED’s Shannon entropy and Rényi entropy [30]. The specific algorithm is presented in Table 1.

**Table 1:** Algorithm for constructing Boot-t CIs.

Algorithm:	Constructing Boot-t CIs.
**1**	Setting the number of simulations B.
**2**	The estimates of *β* and *λ*, denoted as $\hat{\beta }$ and $\hat{\lambda }$, respectively, are obtained from the original PC-II data $\underline{x}=(x_{1:m:n},x_{2:m:n},\cdots ,x_{m:m:n})$.
**3**	Keeping the censoring schemes unchanged, substituting $\hat{\beta }$ and $\hat{\lambda }$ into the distribution function of the GIED, we obtain the bootstrap samples x*. From x*, we derive the bootstrap ML estimates ${\hat{\beta }}^{*}$ and ${\hat{\lambda }}^{*}$. Substituting ${\hat{\beta }}^{*}$ and ${\hat{\lambda }}^{*}$ into Eqs (10) and (11), we obtain ${\hat{H}}_{S}^{*}$ and ${\hat{H}}_{R}^{*}$, along with their variances $Var({\hat{H}}_{S}^{*})$ and $Var({\hat{H}}_{R}^{*})$.
**4**	Calculate the corresponding statistics $\delta =\sqrt{B}({\hat{H}}_{S}^{*}-{\hat{H}}_{S})/\sqrt{Var({\hat{H}}_{S}^{*})}$ and $\sigma =\sqrt{B}({\hat{H}}_{R}^{*}-{\hat{H}}_{R})/\sqrt{Var({\hat{H}}_{R}^{*})}$, as well as the CDFs *W*_*s*_(*x*) = *P*(*δ*≤*x*) and *W*_*R*_(*x*) = *P*(*σ*≤*x*) corresponding to the statistics, based on the obtained ${\hat{H}}_{S}^{*}$ and ${\hat{H}}_{R}^{*}$.
**5**	Repeat steps 3 and 4 for a total of B times, resulting in a series of statistics (*δ*_1_,*δ*_2_,⋯,*δ*_*m*_) and (*σ*_1_,*σ*_2_,⋯,*σ*_*m*_).
**6**	The series of statistics obtained are sorted in ascending order to obtain (*δ*_(1)_,*δ*_(2)_,⋯,*δ*_(*m*)_) and (*σ*_(1)_,*σ*_(2)_,⋯,*σ*_(*m*)_).

Provide the definition:

$${\hat{H}}_{BS}(x)={\hat{H}}_{S}+B^{-\frac{1}{2}}\sqrt{Var({\hat{H}}_{S})}W_{S}^{-1}(x),\;{\hat{H}}_{BR}(x)={\hat{H}}_{R}+B^{-\frac{1}{2}}\sqrt{Var({\hat{H}}_{R})}W_{R}^{-1}(x).$$

Therefore, the 100(1−*θ*)% Boot-t CIs for Shannon entropy and Rényi entropy are $\left({\hat{H}}_{BS}(\frac{\theta }{2}),{\hat{H}}_{BS}(1-\frac{\theta }{2})\right)$ and $\left({\hat{H}}_{BR}(\frac{\theta }{2}),{\hat{H}}_{BR}(1-\frac{\theta }{2})\right)$, respectively. The variance of the entropy can be obtained from the Fisher matrix. Next, we will calculate the variance of the entropy:

Construct the Fisher matrix:

$$I(\hat{\beta },\hat{\lambda })={\left[\begin{array}{cc} -\frac{\partial^{2}l(\beta ,\lambda )}{\partial \beta^{2}} & -\frac{\partial^{2}l(\beta ,\lambda )}{\partial \beta \partial \lambda }\\ -\frac{\partial^{2}l(\beta ,\lambda )}{\partial \lambda \partial \beta } & -\frac{\partial^{2}l(\beta ,\lambda )}{\partial \lambda^{2}} \end{array}\right]}_{\beta =\hat{\beta },\lambda =\hat{\lambda }},$$

where:

$$\frac{\partial^{2}l(\beta ,\lambda )}{\partial \beta^{2}}=-\frac{m}{\beta^{2}},\;\frac{\partial^{2}l(\beta ,\lambda )}{\partial \lambda^{2}}=-\frac{m}{\lambda^{2}}-\sum_{i=1}^{m}(\beta R_{i}+\beta -1)\frac{e^{-\frac{\lambda }{x_{i}}}}{x_{i}^{2}{\left(1-e^{-\frac{\lambda }{x_{i}}}\right)}^{2}},$$

$$\frac{\partial^{2}l(\beta ,\lambda )}{\partial \beta \partial \lambda }=\sum_{i=1}^{m}(R_{i}+1)\frac{e^{-\frac{\lambda }{x_{i}}}}{x_{i}(1-e^{-\frac{\lambda }{x_{i}}})}=\frac{\partial^{2}l(\beta ,\lambda )}{\partial \lambda \partial \beta }.$$

We can obtain the inverse matrix of the Fisher matrix, denoted as $I^{-1}(\hat{\beta },\hat{\lambda })$. Since taking derivatives directly with respect to *β* and *λ* in Eqs (5) and (6) can be quite complicated, we rewrite Eqs (5) and (6) as follows, it should be noted that, for the sake of simplicity, we make $u(\lambda ,x)=1-e^{-\lambda /x}$.


$$H_{S}(f)=-\ln(\lambda \beta )+2\int_{0}^{\infty }\frac{\lambda \beta }{x^{2}}(1-u)u^{\beta -1}\ln\;xdx+\psi (\beta +1)-\psi (1)+\frac{\beta -1}{\beta },$$ (12)

$$H_{R}(f)=\frac{\alpha \ln(\lambda \beta )}{1-\alpha }+\frac{\ln\int_{0}^{\infty }x^{-2\alpha }{\left(1-u\right)}^{\alpha }u^{\alpha (\beta -1)}dx}{1-\alpha },$$ (13)

Therefore, from Eqs (12) and (13), we have:

$$\frac{\partial H_{S}}{\partial \beta }=-\frac{1}{\beta }+2\int_{0}^{\infty }\frac{\lambda }{x^{2}}(1-u)u^{\beta -1}\ln\;x(1+\beta \ln\;u)dx+\psi^{\prime }(\beta +1)+\frac{1}{\beta^{2}},$$

$$\frac{\partial H_{S}}{\partial \lambda }=-\frac{1}{\lambda }+2\int_{0}^{\infty }\frac{\beta }{x^{3}}(1-u)u^{\beta -2}\ln\;x(xu+\lambda \beta -\lambda \beta u-\lambda )dx,$$

$$\frac{\partial H_{R}}{\partial \beta }=\frac{\alpha }{\beta (1-\alpha )}+\frac{\alpha \int_{0}^{\infty }x^{-2\alpha }{\left(1-u\right)}^{\alpha }u^{\alpha (\beta -1)}\ln\;udx}{(1-\alpha )\int_{0}^{\infty }x^{-2\alpha }{\left(1-u\right)}^{\alpha }u^{\alpha (\beta -1)}dx},$$

$$\frac{\partial H_{R}}{\partial \lambda }=\frac{\alpha }{\lambda (1-\alpha )}-\frac{\alpha \int_{0}^{\infty }x^{-2\alpha -1}{\left(1-u\right)}^{\alpha }u^{\alpha (\beta -1)-1}(\beta u-\beta +1)dx}{(1-\alpha )\int_{0}^{\infty }x^{-2\alpha }{\left(1-u\right)}^{\alpha }u^{\alpha (\beta -1)}dx}.$$

Therefore

$$Var({\hat{H}}_{S})={T_{S}I^{-1}(\beta ,\lambda )T_{S}^{\prime }|}_{\beta =\hat{\beta },\lambda =\hat{\lambda }},\;Var({\hat{H}}_{R})={T_{R}I^{-1}(\beta ,\lambda )T_{R}^{\prime }|}_{\beta =\hat{\beta },\lambda =\hat{\lambda }},$$

where $T_{S}^{\prime }$ represents the transpose of *T*_*S*_ and $T_{R}^{\prime }$ represents the transpose of *T*_*R*_. Definition $T_{S}=(\frac{\partial H_{S}}{\partial \beta },\frac{\partial H_{S}}{\partial \lambda }),T_{R}=(\frac{\partial H_{R}}{\partial \beta },\frac{\partial H_{R}}{\partial \lambda })$.

## 4. Bayesian estimation of entropy

Bayesian estimation is a parameter estimation method based on Bayes’ theorem in statistics [31]. It utilizes prior information and sample data to obtain a posterior probability distribution by updating the prior probability distribution, and infers parameter estimates and uncertainties from it. Bayesian estimation, as a common statistical inference method, has the advantage of flexibly combining prior knowledge and observation data to provide more accurate inference results. It is widely used in many fields to deal with uncertainty, model selection, and prediction, such as statistics, machine learning, image and signal processing, financial risk assessment, and other fields [32–34]. In this section, we propose a posterior density function for GIED based on PC-II samples, and use Bayesian estimation to obtain the estimation results of Shannon entropy and Rényi entropy under three loss functions: LLF, ELF, and DLF.

### 4.1. Conditional posterior distribution

Based on the principle of Bayesian estimation, selecting an appropriate prior distribution is the core step of Bayesian estimation, which allows for the fusion of previous knowledge, experience, or data into parameter inference, thereby providing more accurate, reliable, and comprehensive parameter estimation results. In this section, we use information priors for inference. The selection of information priors aims to utilize previous knowledge, experience, or data to assist in parameter inference in the Bayesian estimation process. Compared to non-informative prior, informative prior has more guidance and accuracy, thereby improving the reliability and accuracy of parameter es-timation results [35]. In this study, we adopt the gamma distribution as the prior distribution due to its flexibility and conjugate nature, which allows us to obtain a simplified posterior distribution when combined with the LF. This simplification greatly facilitates the process of Bayesian estimation. Assuming *β* and *λ* are independent random variables following Γ(*η*_1_,*γ*_1_) and Γ(*η*_2_,*γ*_2_) distributions, respectively, the density functions of *β* and *λ* can be represented as follows:

$$\pi (\beta |\eta_{1},\gamma_{1})=\frac{{\gamma_{1}}^{\eta_{1}}}{\Gamma (\eta_{1})}\beta^{\eta_{1}-1}e^{-\gamma_{1}\beta };\;\eta_{1}>0,\gamma_{1}>0,$$

$$\pi (\lambda |\eta_{2},\gamma_{2})=\frac{{\gamma_{2}}^{\eta_{2}}}{\Gamma (\eta_{2})}\lambda^{\eta_{2}-1}e^{-\gamma_{2}\lambda };\;\eta_{2}>0,\gamma_{2}>0.$$

The joint prior on *β* and *λ* is given by:

$$\pi \left(\beta ,\lambda \right)=\frac{{\gamma_{1}}^{\eta_{1}}{\gamma_{2}}^{\eta_{2}}}{\Gamma (\eta_{1})\Gamma (\eta_{2})}\beta^{\eta_{1}-1}\lambda^{\eta_{2}-1}e^{-\gamma_{1}\beta -\gamma_{2}\lambda }.$$

By applying Bayesian theorem, the joint posterior density of *β* and *λ* can be determined as:

$$\pi (\beta ,\lambda |\underline{x})=\frac{L(\beta ,\lambda |\underline{x})\pi (\beta ,\lambda )}{\int_{0}^{\infty }\int_{0}^{\infty }L(\beta ,\lambda |\underline{x})\pi (\beta ,\lambda )d\beta d\lambda },$$

where $\underline{x}=(x_{1:m:n},x_{2:m:n},\cdots ,x_{m:m:n})$.

Bayesian estimation is a parameter estimation method based on Bayes’ theorem. In the decision-making process, we often need to consider the risks and losses associated with different decisions. Introducing a loss function can help quantify the risks associated with different decisions during the estimation process to find the optimal decision. In this section, we introduce three loss functions: LLF, ELF, and DLF. LLF is an asymmetric loss function proposed, which combines the characteristics of exponential loss and linear loss and allows balancing the response to prediction errors based on parameter adjustments. ELF is a loss function used for classification problems, based on the concept of information entropy, to measure the difference between model predictions and true values. DLF is a common asymmetric loss function proposed by DeGroot [36], often used in Bayesian decision theory to compare different decision strategies. Compared to LLF and ELF, DLF computation is relatively simple and easier to implement. Therefore, we choose three loss functions, LLF, ELF and DLF, to comprehensively consider factors such as prediction error, information content and practicality, and hope to find the most suitable loss function for GIED model entropy estimation through comparative analysis. The BEs of these different loss functions is provided below [37–39] (See Table 2).

**Table 2:** BEs with different loss functions.

Loss Function	Expression	BE
**LLF**	$e^{c[\hat{H}-H]}-c[\hat{H}-H]-1$	$-\frac{1}{c}\ln E[e^{-cH}|\underline{x}]$
**ELF**	$\frac{\hat{H}}{H}-\ln(\frac{\hat{H}}{H})-1$	${\left[E[H^{-1}|\underline{x}]\right]}^{-1}$
**DLF**	${\left(\frac{\hat{H}-H}{\hat{H}}\right)}^{2}$	$\frac{E[H^{2}|\underline{x}]}{E[H|\underline{x}]}$

Here, *H* represents the entropy function, and $\hat{H}$ represents the estimates of *H*, *c* represents the hyperparameter of LLF.

(1) Under the LLF, the BEs of Shannon entropy and Rényi entropy are as follows:

$${\hat{H}}_{SL}=-\frac{1}{c}\ln E[e^{-cH_{S}}|\underline{x}]=-\frac{1}{c}\ln[\int_{0}^{\infty }\int_{0}^{\infty }e^{-cH_{S}}\pi (\beta ,\lambda |\underline{x})d\beta d\lambda ],$$ (14)

$${\hat{H}}_{RL}=-\frac{1}{c}\ln E[e^{-cH_{R}}|\underline{x}]=-\frac{1}{c}\ln[\int_{0}^{\infty }\int_{0}^{\infty }e^{-cH_{R}}\pi (\beta ,\lambda |\underline{x})d\beta d\lambda ].$$ (15)

(2) Under the ELF, the BEs of Shannon entropy and Rényi entropy are as follows:

$${\hat{H}}_{SE}={\left[E[H_{S}^{-1}|\underline{x}]\right]}^{-1}={\left(\int_{0}^{\infty }\int_{0}^{\infty }H_{S}^{-1}\pi (\beta ,\lambda |\underline{x})d\beta d\lambda \right)}^{-1},$$ (16)

$${\hat{H}}_{RE}={\left[E[H_{R}^{-1}|\underline{x}]\right]}^{-1}={\left(\int_{0}^{\infty }\int_{0}^{\infty }H_{R}^{-1}\pi (\beta ,\lambda |\underline{x})d\beta d\lambda \right)}^{-1}.$$ (17)

(3) Under the DLF, the BEs of Shannon entropy and Rényi entropy are as follows:

$${\hat{H}}_{SD}=\frac{E[H_{S}^{2}|\underline{x}]}{E[H_{S}|\underline{x}]}=\frac{\int_{0}^{\infty }\int_{0}^{\infty }H_{S}^{2}\pi (\beta ,\lambda |\underline{x})d\beta d\lambda }{\int_{0}^{\infty }\int_{0}^{\infty }H_{S}\pi (\beta ,\lambda |\underline{x})d\beta d\lambda },$$ (18)

$${\hat{H}}_{RD}=\frac{E[H_{R}^{2}|\underline{x}]}{E[H_{R}|\underline{x}]}=\frac{\int_{0}^{\infty }\int_{0}^{\infty }H_{R}^{2}\pi (\beta ,\lambda |\underline{x})d\beta d\lambda }{\int_{0}^{\infty }\int_{0}^{\infty }H_{R}\pi (\beta ,\lambda |\underline{x})d\beta d\lambda }.$$ (19)

Indeed, the aforementioned BEs are in the form of a ratio of double integrals, and they are not in explicit form, making it difficult to compute the results directly. Therefore, we can employ the Lindley approximation algorithm to compute the BEs of Shannon entropy and Rényi entropy.

### 4.2. Lindley approximation

The Lindley approximation algorithm is a Bayesian statistical inference approximation method proposed by Lindley, used to calculate estimates of parameters. According to Abo-Kasem [23], we provide the Lindley approximate equation:

$$\begin{array}{l} I(\underline{x})=\varphi (\hat{\beta },\hat{\lambda })+\frac{1}{2}[({\hat{\varphi }}_{\beta \beta }+2{\hat{\varphi }}_{\beta }{\hat{\rho }}_{\beta }){\hat{\sigma }}_{\beta \beta }+({\hat{\varphi }}_{\lambda \beta }+2{\hat{\varphi }}_{\lambda }{\hat{\rho }}_{\beta }){\hat{\sigma }}_{\lambda \beta }\\ \;+({\hat{\varphi }}_{\beta \lambda }+2{\hat{\varphi }}_{\beta }{\hat{\rho }}_{\lambda }){\hat{\sigma }}_{\beta \lambda }+({\hat{\varphi }}_{\lambda \lambda }+2{\hat{\varphi }}_{\lambda }{\hat{\rho }}_{\lambda }){\hat{\sigma }}_{\lambda \lambda }]\\ \;+\frac{1}{2}[({\hat{\varphi }}_{\beta }{\hat{\sigma }}_{\beta \beta }+{\hat{\varphi }}_{\lambda }{\hat{\sigma }}_{\beta \lambda })({\hat{l}}_{\beta \beta \beta }{\hat{\sigma }}_{\beta \beta }+{\hat{l}}_{\beta \lambda \beta }{\hat{\sigma }}_{\beta \lambda }+{\hat{l}}_{\lambda \beta \beta }{\hat{\sigma }}_{\lambda \beta }+{\hat{l}}_{\lambda \lambda \beta }{\hat{\sigma }}_{\lambda \lambda })\\ \;+({\hat{\varphi }}_{\beta }{\hat{\sigma }}_{\lambda \beta }+{\hat{\varphi }}_{\lambda }{\hat{\sigma }}_{\lambda \lambda })({\hat{l}}_{\lambda \beta \beta }{\hat{\sigma }}_{\beta \beta }+{\hat{l}}_{\beta \lambda \lambda }{\hat{\sigma }}_{\beta \lambda }+{\hat{l}}_{\lambda \beta \lambda }{\hat{\sigma }}_{\lambda \beta }+{\hat{l}}_{\lambda \lambda \lambda }{\hat{\sigma }}_{\lambda \lambda })], \end{array}$$ (20)

where *φ*(*β*,*λ*) is a function of *β* and *λ*. *ρ*(*β*,*λ*) is the logarithm of the joint prior distribution of *β* and *λ*, that is ρ(β,λ)=lnπ(β,λ). *l*(*β*,*λ*|*x*) is the logarithm of the LF. And $\hat{\beta }$ and $\hat{\lambda }$ are the ML estimates of *β* and *λ*, and the subscripts denote the partial derivatives of the variables, such as, *φ*_*β*_ is the first-order derivative of *β* in *φ*(*β*,*λ*). Similarly, the others are denoted as follows:

$${\hat{\rho }}_{\beta }=\frac{\eta_{1}-1}{\hat{\beta }}-\gamma_{1},\;{\hat{\rho }}_{\lambda }=\frac{\eta_{2}-1}{\hat{\lambda }}-\gamma_{2},$$

$${\hat{l}}_{\beta \beta \beta }={\frac{\partial^{3}l}{\partial \beta^{3}}|}_{\beta =\hat{\beta }}=\frac{2m}{\beta^{3}},\;{\hat{l}}_{\lambda \beta \beta }={\frac{\partial^{3}l}{\partial \lambda \partial \beta^{2}}|}_{\beta =\hat{\beta },\lambda =\hat{\lambda }}=0={\hat{l}}_{\beta \lambda \beta },$$

$${\hat{l}}_{\lambda \lambda \beta }={\frac{\partial^{3}l}{\partial \lambda^{2}\partial \beta }|}_{\beta =\hat{\beta },\lambda =\hat{\lambda }}=-\sum_{i=1}^{m}(R_{i}+1)\frac{e^{-\frac{\hat{\lambda }}{x_{i}}}}{x_{i}^{2}{\left(1-e^{-\frac{\hat{\lambda }}{x_{i}}}\right)}^{2}}={\hat{l}}_{\beta \lambda \lambda }={\hat{l}}_{\lambda \beta \lambda },$$

$${\hat{l}}_{\lambda \lambda \lambda }={\frac{\partial^{3}l}{\partial \lambda^{3}}|}_{\lambda =\hat{\lambda }}=\frac{2m}{{\hat{\lambda }}^{3}}+\sum_{i=1}^{m}(\hat{\beta }R_{i}+\hat{\beta }-1)\frac{e^{-\frac{\hat{\lambda }}{x_{i}}}(1+e^{-\frac{\hat{\lambda }}{x_{i}}})}{x_{i}^{3}{\left(1-e^{-\frac{\hat{\lambda }}{x_{i}}}\right)}^{3}}.$$

Based on the given equations, the following representation holds for Eqs (14)–(19):

(1) Computing the BE of Shannon entropy under LLF:

In that case, we have $\varphi_{S}(\beta ,\lambda )=e^{-cH_{S}}$, thus

$$\varphi_{S\beta }=-c\;e^{-cH_{S}}[-\frac{1}{\beta }+2\int_{0}^{\infty }\frac{\lambda }{x^{2}}(1-u)u^{\beta -1}\ln\;x(1+\beta \ln\;u)dx+\psi^{\prime }(\beta +1)+\frac{1}{\beta^{2}}],$$

$$\begin{array}{l} \varphi_{S\beta \beta }=c^{2}\;e^{-cH_{S}}[-\frac{1}{\beta }+2\int_{0}^{\infty }\frac{\lambda }{x^{2}}(1-u)u^{\beta -1}\ln\;x(1+\beta \ln\;u)dx+\psi^{\prime }(\beta +1)+\frac{1}{\beta^{2}}{]}^{2}\\ \;-c\;e^{-cH_{S}}[\frac{1}{\beta^{2}}+2\int_{0}^{\infty }\frac{\lambda }{x^{2}}(1-u)u^{\beta -1}\ln\;u\ln\;x(2+\beta \ln\;u)dx+\psi^{\prime \prime }(\beta +1)-\frac{2}{\beta^{3}}], \end{array}$$

$$\varphi_{S\lambda }=-c\;e^{-cH_{S}}[-\frac{1}{\lambda }+2\int_{0}^{\infty }\frac{\beta }{x^{3}}(1-u)u^{\beta -2}\ln\;x(xu+\lambda \beta -\lambda \beta u-\lambda )dx],$$

$$\begin{array}{l} \varphi_{S\lambda \lambda }=c^{2}\;e^{-cH_{S}}[-\frac{1}{\lambda }+2\int_{0}^{\infty }\frac{\beta }{x^{3}}(1-u)u^{\beta -2}\ln\;x(xu+\lambda \beta -\lambda \beta u-\lambda )dx{]}^{2}\\ \;-c\;e^{-cH_{S}}\{\frac{1}{\lambda^{2}}-2\int_{0}^{\infty }\frac{\beta }{x^{4}}(1-u)u^{\beta -2}\ln\;x(xu+\lambda \beta -\lambda \beta u-\lambda )[1-(\beta -2)(1-u)u^{-1}]dx\\ \;+2\int_{0}^{\infty }\frac{\beta }{x^{3}}(1-u)u^{\beta -2}\ln\;x[1-u+\beta -\beta u-\lambda \beta (1-u)x^{-1}-1]dx\}, \end{array}$$

$$\begin{array}{l} \varphi_{S\beta \lambda }=\varphi_{S\lambda \beta }=c^{2}\;e^{-cH_{S}}[-\frac{1}{\lambda }+2\int_{0}^{\infty }\frac{\beta }{x^{3}}(1-u)u^{\beta -2}\ln\;x(xu+\lambda \beta -\lambda \beta u-\lambda )dx]\\ \;\times [-\frac{1}{\beta }+2\int_{0}^{\infty }\frac{\lambda }{x^{2}}(1-u)u^{\beta -1}\ln\;x(1+\beta \ln\;u)dx+\psi^{\prime }(\beta +1)+\frac{1}{\beta^{2}}]\\ \;-c\;e^{-cH_{S}}[2\int_{0}^{\infty }\frac{1}{x^{2}}(1-u)u^{\beta -1}\ln\;x(1+\beta \ln\;u)(1-\frac{\lambda }{x})dx\\ \;+2\int_{0}^{\infty }\frac{\lambda }{x^{3}}(1-u{)}^{2}u^{\beta -2}\ln\;x(2\beta +\beta^{2}\ln\;u-\beta \ln\;u-1)dx]. \end{array}$$

Substitute the above equations into Eq (20) to obtain $E[e^{-cH_{S}}|\underline{x}]$, and then the BE of Shannon entropy under LLF can be obtained from Eq (14).

(2) Computing the BE of Rényi entropy under LLF:

In that case, we have $\varphi_{R}(\beta ,\lambda )=e^{-cH_{R}}$, thus

$$\varphi_{R\beta }=-c\;e^{-cH_{R}}[\frac{\alpha }{\beta (1-\alpha )}+\frac{\alpha \int_{0}^{\infty }x^{-2\alpha }{\left(1-u\right)}^{\alpha }u^{\alpha (\beta -1)}\ln\;udx}{(1-\alpha )\int_{0}^{\infty }x^{-2\alpha }{\left(1-u\right)}^{\alpha }u^{\alpha (\beta -1)}dx}],$$

$$\begin{array}{l} \varphi_{R\beta \beta }=c^{2}\;e^{-cH_{R}}[\frac{\alpha }{\beta (1-\alpha )}+\frac{\alpha \int_{0}^{\infty }x^{-2\alpha }{\left(1-u\right)}^{\alpha }u^{\alpha (\beta -1)}\ln\;udx}{(1-\alpha )\int_{0}^{\infty }x^{-2\alpha }{\left(1-u\right)}^{\alpha }u^{\alpha (\beta -1)}dx}{]}^{2}\\ \;-c\;e^{-cH_{R}}\{-\frac{\alpha }{\beta^{2}(1-\alpha )}+\frac{\alpha^{2}\int_{0}^{\infty }x^{-2\alpha }{\left(1-u\right)}^{\alpha }u^{\alpha (\beta -1)}{\left(\ln\;u\right)}^{2}dx}{(1-\alpha )\int_{0}^{\infty }x^{-2\alpha }{\left(1-u\right)}^{\alpha }u^{\alpha (\beta -1)}dx}\\ \;-\frac{\alpha^{2}[\int_{0}^{\infty }x^{-2\alpha }{\left(1-u\right)}^{\alpha }u^{\alpha (\beta -1)}\ln\;udx{]}^{2}}{(1-\alpha )[\int_{0}^{\infty }x^{-2\alpha }{\left(1-u\right)}^{\alpha }u^{\alpha (\beta -1)}dx{]}^{2}}\}, \end{array}$$

$$\varphi_{R\lambda }=-c\;e^{-cH_{R}(f)}[\frac{\alpha }{\lambda (1-\alpha )}-\frac{\alpha \int_{0}^{\infty }x^{-2\alpha -1}{\left(1-u\right)}^{\alpha }u^{\alpha (\beta -1)-1}(\beta u-\beta +1)dx}{(1-\alpha )\int_{0}^{\infty }x^{-2\alpha }{\left(1-u\right)}^{\alpha }u^{\alpha (\beta -1)}dx}],$$

$$\begin{array}{l} \varphi_{R\lambda \lambda }=c^{2}\;e^{-cH_{R}(f)}[\frac{\alpha }{\lambda (1-\alpha )}-\frac{\alpha \int_{0}^{\infty }x^{-2\alpha -1}{\left(1-u\right)}^{\alpha }u^{\alpha (\beta -1)-1}(\beta u-\beta +1)dx}{(1-\alpha )\int_{0}^{\infty }x^{-2\alpha }{\left(1-u\right)}^{\alpha }u^{\alpha (\beta -1)}dx}{]}^{2}\\ \;-c\;e^{-cH_{R}(f)}\{-\frac{\alpha }{\lambda^{2}(1-\alpha )}+\frac{\alpha \int_{0}^{\infty }x^{-2\alpha -2}{\left(1-u\right)}^{\alpha }u^{\alpha (\beta -1)-2}(\beta u-\beta +1)(\alpha \beta u-\alpha \beta +\alpha -u+1)dx}{(1-\alpha )\int_{0}^{\infty }x^{-2\alpha }{\left(1-u\right)}^{\alpha }u^{\alpha (\beta -1)}dx}\\ \;-\frac{\alpha \beta \int_{0}^{\infty }x^{-2\alpha -2}{\left(1-u\right)}^{\alpha +1}u^{\alpha (\beta -1)-1}dx}{(1-\alpha )\int_{0}^{\infty }x^{-2\alpha }{\left(1-u\right)}^{\alpha }u^{\alpha (\beta -1)}dx}-\frac{\alpha^{2}[\int_{0}^{\infty }x^{-2\alpha -1}{\left(1-u\right)}^{\alpha }u^{\alpha (\beta -1)-1}(\beta u-\beta +1)dx{]}^{2}}{(1-\alpha )[\int_{0}^{\infty }x^{-2\alpha }{\left(1-u\right)}^{\alpha }u^{\alpha (\beta -1)}dx{]}^{2}}\}, \end{array}$$

$$\begin{array}{l} \varphi_{R\beta \lambda }=\varphi_{R\lambda \beta }=c^{2}\;e^{-cH_{R}(f)}[\frac{\alpha }{\lambda (1-\alpha )}-\frac{\alpha \int_{0}^{\infty }x^{-2\alpha -1}{\left(1-u\right)}^{\alpha }u^{\alpha (\beta -1)-1}(\beta u-\beta +1)dx}{(1-\alpha )\int_{0}^{\infty }x^{-2\alpha }{\left(1-u\right)}^{\alpha }u^{\alpha (\beta -1)}dx}]\\ \;\times [\frac{\alpha }{\beta (1-\alpha )}+\frac{\alpha \int_{0}^{\infty }x^{-2\alpha }{\left(1-u\right)}^{\alpha }u^{\alpha (\beta -1)}\ln\;udx}{(1-\alpha )\int_{0}^{\infty }x^{-2\alpha }{\left(1-u\right)}^{\alpha }u^{\alpha (\beta -1)}dx}]\\ \;-c\;e^{-cH_{R}(f)}\{-\frac{\alpha^{2}\int_{0}^{\infty }x^{-2\alpha -1}{\left(1-u\right)}^{\alpha }u^{\alpha (\beta -1)-1}\ln\;u(\beta u-\beta +1)dx}{(1-\alpha )\int_{0}^{\infty }x^{-2\alpha }{\left(1-u\right)}^{\alpha }u^{\alpha (\beta -1)}dx}\\ \;+\frac{\alpha \int_{0}^{\infty }x^{-2\alpha -1}{\left(1-u\right)}^{\alpha +1}u^{\alpha (\beta -1)-1}dx}{(1-\alpha )\int_{0}^{\infty }x^{-2\alpha }{\left(1-u\right)}^{\alpha }u^{\alpha (\beta -1)}dx}\\ \;+\frac{\alpha^{2}\int_{0}^{\infty }x^{-2\alpha }{\left(1-u\right)}^{\alpha }u^{\alpha (\beta -1)}\ln\;udx\int_{0}^{\infty }x^{-2\alpha -1}{\left(1-u\right)}^{\alpha }u^{\alpha (\beta -1)-1}(\beta u-\beta +1)dx}{(1-\alpha )[\int_{0}^{\infty }x^{-2\alpha }{\left(1-u\right)}^{\alpha }u^{\alpha (\beta -1)}dx{]}^{2}}\}. \end{array}$$

Substitute the above equations into Eq (20) to obtain $E[e^{-cH_{R}}|\underline{x}]$, and then the BE of Rényi entropy under LLF can be obtained from Eq (15).

(3) Computing the BE of Shannon entropy under ELF:

In that case, we have $\varphi_{S}(\beta ,\lambda )=H_{S}^{-1}$,thus

$$\varphi_{S\beta }=-{H_{S}}^{-2}[-\frac{1}{\beta }+2\int_{0}^{\infty }\frac{\lambda }{x^{2}}(1-u)u^{\beta -1}\ln\;x(1+\beta \ln\;u)dx+\psi^{\prime }(\beta +1)+\frac{1}{\beta^{2}}],$$

$$\begin{array}{l} \varphi_{S\beta \beta }=2{H_{S}}^{-3}[-\frac{1}{\beta }+2\int_{0}^{\infty }\frac{\lambda }{x^{2}}(1-u)u^{\beta -1}\ln\;x(1+\beta \ln\;u)dx+\psi^{\prime }(\beta +1)+\frac{1}{\beta^{2}}{]}^{2}\\ \;-{H_{S}}^{-2}[\frac{1}{\beta^{2}}+2\int_{0}^{\infty }\frac{\lambda }{x^{2}}(1-u)u^{\beta -1}\ln\;u\ln\;x(2+\beta \ln\;u)dx+\psi^{\prime \prime }(\beta +1)-\frac{2}{\beta^{3}}], \end{array}$$

$$\varphi_{S\lambda }=-{H_{S}}^{-2}[-\frac{1}{\lambda }+2\int_{0}^{\infty }\frac{\beta }{x^{3}}(1-u)u^{\beta -2}\ln\;x(xu+\lambda \beta -\lambda \beta u-\lambda )dx],$$

$$\begin{array}{l} \varphi_{S\lambda \lambda }=2{H_{S}}^{-3}[-\frac{1}{\lambda }+2\int_{0}^{\infty }\frac{\beta }{x^{3}}(1-u)u^{\beta -2}\ln\;x(xu+\lambda \beta -\lambda \beta u-\lambda )dx{]}^{2}\\ \;-{H_{S}}^{-2}\{\frac{1}{\lambda^{2}}-2\int_{0}^{\infty }\frac{\beta }{x^{4}}(1-u)u^{\beta -2}\ln\;x(xu+\lambda \beta -\lambda \beta u-\lambda )[1-(\beta -2)(1-u)u^{-1}]dx\\ \;+2\int_{0}^{\infty }\frac{\beta }{x^{3}}(1-u)u^{\beta -2}\ln\;x[1-u+\beta -\beta u-\lambda \beta (1-u)x^{-1}-1]dx\}, \end{array}$$

$$\begin{array}{l} \varphi_{S\beta \lambda }=\varphi_{S\lambda \beta }=2{H_{S}}^{-3}[-\frac{1}{\lambda }+2\int_{0}^{\infty }\frac{\beta }{x^{3}}(1-u)u^{\beta -2}\ln\;x(xu+\lambda \beta -\lambda \beta u-\lambda )dx]\\ \;\times [-\frac{1}{\beta }+2\int_{0}^{\infty }\frac{\lambda }{x^{2}}(1-u)u^{\beta -1}\ln\;x(1+\beta \ln\;u)dx+\psi^{\prime }(\beta +1)+\frac{1}{\beta^{2}}]\\ \;-{H_{S}}^{-2}[2\int_{0}^{\infty }\frac{1}{x^{2}}(1-u)u^{\beta -1}\ln\;x(1+\beta \ln\;u)(1-\frac{\lambda }{x})dx\\ \;+2\int_{0}^{\infty }\frac{\lambda }{x^{3}}(1-u{)}^{2}u^{\beta -2}\ln\;x(2\beta +\beta^{2}\ln\;u-\beta \ln\;u-1)dx]. \end{array}$$

Substitute the above equations into Eq (20) to obtain $E[H_{S}^{-1}|\underline{x}]$, and then the BE of Shannon entropy under ELF can be obtained from Eq (16).

(4) Computing the BE of Rényi entropy under ELF:

In that case, we have $\varphi_{R}(\beta ,\lambda )=H_{R}^{-1}$, thus

$$\varphi_{R\beta }=-{H_{R}}^{-2}[\frac{\alpha }{\beta (1-\alpha )}+\frac{\alpha \int_{0}^{\infty }x^{-2\alpha }{\left(1-u\right)}^{\alpha }u^{\alpha (\beta -1)}\ln\;udx}{(1-\alpha )\int_{0}^{\infty }x^{-2\alpha }{\left(1-u\right)}^{\alpha }u^{\alpha (\beta -1)}dx}],$$

$$\begin{array}{l} \varphi_{R\beta \beta }=2{H_{R}}^{-3}[\frac{\alpha }{\beta (1-\alpha )}+\frac{\alpha \int_{0}^{\infty }x^{-2\alpha }{\left(1-u\right)}^{\alpha }u^{\alpha (\beta -1)}\ln\;udx}{(1-\alpha )\int_{0}^{\infty }x^{-2\alpha }{\left(1-u\right)}^{\alpha }u^{\alpha (\beta -1)}dx}{]}^{2}\\ \;-{H_{R}}^{-2}\{-\frac{\alpha }{\beta^{2}(1-\alpha )}+\frac{\alpha^{2}\int_{0}^{\infty }x^{-2\alpha }{\left(1-u\right)}^{\alpha }u^{\alpha (\beta -1)}{\left(\ln\;u\right)}^{2}dx}{(1-\alpha )\int_{0}^{\infty }x^{-2\alpha }{\left(1-u\right)}^{\alpha }u^{\alpha (\beta -1)}dx}\\ \;-\frac{\alpha^{2}{\left[\int_{0}^{\infty }x^{-2\alpha }{\left(1-u\right)}^{\alpha }u^{\alpha (\beta -1)}\ln\;udx\right]}^{2}}{(1-\alpha ){\left[\int_{0}^{\infty }x^{-2\alpha }{\left(1-u\right)}^{\alpha }u^{\alpha (\beta -1)}dx\right]}^{2}}\}, \end{array}$$

$$\varphi_{R\lambda }=-{H_{R}}^{-2}[\frac{\alpha }{\lambda (1-\alpha )}-\frac{\alpha \int_{0}^{\infty }x^{-2\alpha -1}{\left(1-u\right)}^{\alpha }u^{\alpha (\beta -1)-1}(\beta u-\beta +1)dx}{(1-\alpha )\int_{0}^{\infty }x^{-2\alpha }{\left(1-u\right)}^{\alpha }u^{\alpha (\beta -1)}dx}],$$

$$\begin{array}{l} \varphi_{R\lambda \lambda }=2{H_{R}}^{-3}[\frac{\alpha }{\lambda (1-\alpha )}-\frac{\alpha \int_{0}^{\infty }x^{-2\alpha -1}{\left(1-u\right)}^{\alpha }u^{\alpha (\beta -1)-1}(\beta u-\beta +1)dx}{(1-\alpha )\int_{0}^{\infty }x^{-2\alpha }{\left(1-u\right)}^{\alpha }u^{\alpha (\beta -1)}dx}{]}^{2}\\ \;-{H_{R}}^{-2}[-\frac{\alpha }{\lambda^{2}(1-\alpha )}+\frac{\alpha \int_{0}^{\infty }x^{-2\alpha -2}{\left(1-u\right)}^{\alpha }u^{\alpha (\beta -1)-2}(\beta u-\beta +1)(\alpha \beta u-\alpha \beta +\alpha -u+1)dx}{(1-\alpha )\int_{0}^{\infty }x^{-2\alpha }{\left(1-u\right)}^{\alpha }u^{\alpha (\beta -1)}dx}\\ \;-\frac{\alpha \beta \int_{0}^{\infty }x^{-2\alpha -2}{\left(1-u\right)}^{\alpha +1}u^{\alpha (\beta -1)-1}dx}{(1-\alpha )\int_{0}^{\infty }x^{-2\alpha }{\left(1-u\right)}^{\alpha }u^{\alpha (\beta -1)}dx}-\frac{\alpha^{2}{\left[\int_{0}^{\infty }x^{-2\alpha -1}{\left(1-u\right)}^{\alpha }u^{\alpha (\beta -1)-1}(\beta u-\beta +1)dx\right]}^{2}}{(1-\alpha ){\left[\int_{0}^{\infty }x^{-2\alpha }{\left(1-u\right)}^{\alpha }u^{\alpha (\beta -1)}dx\right]}^{2}}], \end{array}$$

$$\begin{array}{l} \varphi_{R\beta \lambda }=\varphi_{R\lambda \beta }=2{H_{R}}^{-3}[\frac{\alpha }{\lambda (1-\alpha )}-\frac{\alpha \int_{0}^{\infty }x^{-2\alpha -1}{\left(1-u\right)}^{\alpha }u^{\alpha (\beta -1)-1}(\beta u-\beta +1)dx}{(1-\alpha )\int_{0}^{\infty }x^{-2\alpha }{\left(1-u\right)}^{\alpha }u^{\alpha (\beta -1)}dx}]\\ \;\times [\frac{\alpha }{\beta (1-\alpha )}+\frac{\alpha \int_{0}^{\infty }x^{-2\alpha }{\left(1-u\right)}^{\alpha }u^{\alpha (\beta -1)}\ln\;udx}{(1-\alpha )\int_{0}^{\infty }x^{-2\alpha }{\left(1-u\right)}^{\alpha }u^{\alpha (\beta -1)}dx}]\\ \;-{H_{R}}^{-2}\{-\frac{\alpha^{2}\int_{0}^{\infty }x^{-2\alpha -1}{\left(1-u\right)}^{\alpha }u^{\alpha (\beta -1)-1}\ln\;u(\beta u-\beta +1)dx}{(1-\alpha )\int_{0}^{\infty }x^{-2\alpha }{\left(1-u\right)}^{\alpha }u^{\alpha (\beta -1)}dx}\\ \;+\frac{\alpha \int_{0}^{\infty }x^{-2\alpha -1}{\left(1-u\right)}^{\alpha +1}u^{\alpha (\beta -1)-1}dx}{(1-\alpha )\int_{0}^{\infty }x^{-2\alpha }{\left(1-u\right)}^{\alpha }u^{\alpha (\beta -1)}dx}\\ \;+\frac{\alpha^{2}\int_{0}^{\infty }x^{-2\alpha }{\left(1-u\right)}^{\alpha }u^{\alpha (\beta -1)}\ln\;udx\int_{0}^{\infty }x^{-2\alpha -1}{\left(1-u\right)}^{\alpha }u^{\alpha (\beta -1)-1}(\beta u-\beta +1)dx}{(1-\alpha ){\left[\int_{0}^{\infty }x^{-2\alpha }{\left(1-u\right)}^{\alpha }u^{\alpha (\beta -1)}dx\right]}^{2}}\}. \end{array}$$

Substitute the above equations into Eq (20) to obtain $E[H_{R}^{-1}|\underline{x}]$, and then the BE of Rényi entropy under ELF can be obtained from Eq (17).

(5) Computing the BE of Shannon entropy under DLF:

In that case, we have $\varphi_{S}(\beta ,\lambda )=\frac{H_{S}^{2}}{H_{S}}$, letting $\phi_{1}=H_{S}^{2}$, *ϕ*_2_ = *H*_*S*_, thus

$$\phi_{1\beta }=2H_{S}[-\frac{1}{\beta }+2\int_{0}^{\infty }\frac{\lambda }{x^{2}}(1-u)u^{\beta -1}\ln\;x(1+\beta \ln\;u)dx+\psi^{\prime }(\beta +1)+\frac{1}{\beta^{2}}],$$

$$\begin{array}{l} \phi_{1\beta \beta }=2[-\frac{1}{\beta }+2\int_{0}^{\infty }\frac{\lambda }{x^{2}}(1-u)u^{\beta -1}\ln\;x(1+\beta \ln\;u)dx+\psi^{\prime }(\beta +1)+\frac{1}{\beta^{2}}{]}^{2}\\ \;+2H_{S}[\frac{1}{\beta^{2}}+2\int_{0}^{\infty }\frac{\lambda }{x^{2}}(1-u)u^{\beta -1}\ln\;u\ln\;x(2+\beta \ln\;u)dx+\psi^{\prime \prime }(\beta +1)-\frac{2}{\beta^{3}}], \end{array}$$

$$\phi_{1\lambda }=2H_{S}[-\frac{1}{\lambda }+2\int_{0}^{\infty }\frac{\beta }{x^{3}}(1-u)u^{\beta -2}\ln\;x(xu+\lambda \beta -\lambda \beta u-\lambda )dx],$$

$$\begin{array}{l} \phi_{1\lambda \lambda }=2[-\frac{1}{\lambda }+2\int_{0}^{\infty }\frac{\beta }{x^{3}}(1-u)u^{\beta -2}\ln\;x(xu+\lambda \beta -\lambda \beta u-\lambda )dx{]}^{2}\\ \;+2H_{S}\{\frac{1}{\lambda^{2}}-2\int_{0}^{\infty }\frac{\beta }{x^{4}}(1-u)u^{\beta -2}\ln\;x(xu+\lambda \beta -\lambda \beta u-\lambda )[1-(\beta -2)(1-u)u^{-1}]dx\\ \;+2\int_{0}^{\infty }\frac{\beta }{x^{3}}(1-u)u^{\beta -2}\ln\;x[1-u+\beta -\beta u-\lambda \beta (1-u)x^{-1}-1]dx\}, \end{array}$$

$$\begin{array}{l} \phi_{1\beta \lambda }=\phi_{1\lambda \beta }=2[-\frac{1}{\lambda }+2\int_{0}^{\infty }\frac{\beta }{x^{3}}(1-u)u^{\beta -2}\ln\;x(xu+\lambda \beta -\lambda \beta u-\lambda )dx]\\ \;\times [-\frac{1}{\beta }+2\int_{0}^{\infty }\frac{\lambda }{x^{2}}(1-u)u^{\beta -1}\ln\;x(1+\beta \ln\;u)dx+\psi^{\prime }(\beta +1)+\frac{1}{\beta^{2}}]\\ \;+H_{S}\{2\int_{0}^{\infty }\frac{1}{x^{2}}(1-u)u^{\beta -1}\ln\;x(1+\beta \ln\;u)(1-\frac{\lambda }{x})dx\\ \;+2\int_{0}^{\infty }\frac{\lambda }{x^{3}}(1-u{)}^{2}u^{\beta -2}\ln\;x(2\beta +\beta^{2}\ln\;u-\beta \ln\;u-1)dx\}. \end{array}$$

Substitute the above equations into Eq (20) to obtain $E[H_{S}^{2}|\underline{x}]$. Next, we calculate *ϕ*_2_ = *H*_*S*_.


$$\phi_{2\beta }=-\frac{1}{\beta }+2\int_{0}^{\infty }\frac{\lambda }{x^{2}}(1-u)u^{\beta -1}\ln\;x(1+\beta \ln\;u)dx+\psi^{\prime }(\beta +1)+\frac{1}{\beta^{2}},$$

$$\phi_{2\beta \beta }=\frac{1}{\beta^{2}}+2\int_{0}^{\infty }\frac{\lambda }{x^{2}}(1-u)u^{\beta -1}\ln\;u\ln\;x(2+\beta \ln\;u)dx+\psi^{\prime \prime }(\beta +1)-\frac{2}{\beta^{3}},$$

$$\phi_{2\lambda }=-\frac{1}{\lambda }+2\int_{0}^{\infty }\frac{\beta }{x^{3}}(1-u)u^{\beta -2}\ln\;x(xu+\lambda \beta -\lambda \beta u-\lambda )dx,$$

$$\begin{array}{l} \phi_{2\lambda \lambda }=\frac{1}{\lambda^{2}}-2\int_{0}^{\infty }\frac{\beta }{x^{4}}(1-u)u^{\beta -2}\ln\;x(xu+\lambda \beta -\lambda \beta u-\lambda )[1-(\beta -2)(1-u)u^{-1}]dx\\ \;+2\int_{0}^{\infty }\frac{\beta }{x^{3}}(1-u)u^{\beta -2}\ln\;x[1-u+\beta -\beta u-\lambda \beta (1-u)x^{-1}-1]dx, \end{array}$$

$$\begin{array}{l} \phi_{2\beta \lambda }=\phi_{2\lambda \beta }=2\int_{0}^{\infty }\frac{1}{x^{2}}(1-u)u^{\beta -1}\ln\;x(1+\beta \ln\;u)(1-\frac{\lambda }{x})dx\\ \;+2\int_{0}^{\infty }\frac{\lambda }{x^{3}}(1-u{)}^{2}u^{\beta -2}\ln\;x(2\beta +\beta^{2}\ln\;u-\beta \ln\;u-1)dx. \end{array}$$

Substitute the above equations into Eq (20) to obtain *E*[*H*_*S*_|*x*], and then the BE of Shannon entropy under DLF can be obtained from Eq (18).

(6) Computing the BE of Rényi entropy under DLF:

In that case, we have $\varphi_{R}(\beta ,\lambda )=\frac{H_{R}^{2}}{H_{R}}$, letting $\phi_{3}=H_{R}^{2}$, *ϕ*_4_ = *H*_*R*_, thus

$$\phi_{3\beta }=2H_{R}[\frac{\alpha }{\beta (1-\alpha )}+\frac{\alpha \int_{0}^{\infty }x^{-2\alpha }{\left(1-u\right)}^{\alpha }u^{\alpha (\beta -1)}\ln\;udx}{(1-\alpha )\int_{0}^{\infty }x^{-2\alpha }{\left(1-u\right)}^{\alpha }u^{\alpha (\beta -1)}dx}],$$

$$\begin{array}{l} \phi_{3\beta \beta }=2[\frac{\alpha }{\beta (1-\alpha )}+\frac{\alpha \int_{0}^{\infty }x^{-2\alpha }{\left(1-u\right)}^{\alpha }u^{\alpha (\beta -1)}\ln\;udx}{(1-\alpha )\int_{0}^{\infty }x^{-2\alpha }{\left(1-u\right)}^{\alpha }u^{\alpha (\beta -1)}dx}]2\\ \;+2H_{R}(f)\{-\frac{\alpha }{\beta^{2}(1-\alpha )}+\frac{\alpha^{2}\int_{0}^{\infty }x^{-2\alpha }{\left(1-u\right)}^{\alpha }u^{\alpha (\beta -1)}{\left(\ln\;u\right)}^{2}dx}{(1-\alpha )\int_{0}^{\infty }x^{-2\alpha }{\left(1-u\right)}^{\alpha }u^{\alpha (\beta -1)}dx}\\ \;-\frac{\alpha^{2}{\left[\int_{0}^{\infty }x^{-2\alpha }{\left(1-u\right)}^{\alpha }u^{\alpha (\beta -1)}\ln\;udx\right]}^{2}}{(1-\alpha ){\left[\int_{0}^{\infty }x^{-2\alpha }{\left(1-u\right)}^{\alpha }u^{\alpha (\beta -1)}dx\right]}^{2}}\}, \end{array}$$

$$\phi_{3\lambda }=2H_{R}[\frac{\alpha }{\lambda (1-\alpha )}-\frac{\alpha \int_{0}^{\infty }x^{-2\alpha -1}{\left(1-u\right)}^{\alpha }u^{\alpha (\beta -1)-1}(\beta u-\beta +1)dx}{(1-\alpha )\int_{0}^{\infty }x^{-2\alpha }{\left(1-u\right)}^{\alpha }u^{\alpha (\beta -1)}dx}],$$

$$\begin{array}{l} \phi_{3\lambda \lambda }=2[\frac{\alpha }{\lambda (1-\alpha )}-\frac{\alpha \int_{0}^{\infty }x^{-2\alpha -1}{\left(1-u\right)}^{\alpha }u^{\alpha (\beta -1)-1}(\beta u-\beta +1)dx}{(1-\alpha )\int_{0}^{\infty }x^{-2\alpha }{\left(1-u\right)}^{\alpha }u^{\alpha (\beta -1)}dx}{]}^{2}\\ \;+2H_{R}\{-\frac{\alpha }{\lambda^{2}(1-\alpha )}+\frac{\alpha \int_{0}^{\infty }x^{-2\alpha -2}{\left(1-u\right)}^{\alpha }u^{\alpha (\beta -1)-2}(\beta u-\beta +1)(\alpha \beta u-\alpha \beta +\alpha -u+1)dx}{(1-\alpha )\int_{0}^{\infty }x^{-2\alpha }{\left(1-u\right)}^{\alpha }u^{\alpha (\beta -1)}dx}\\ \;-\frac{\alpha \beta \int_{0}^{\infty }x^{-2\alpha -2}{\left(1-u\right)}^{\alpha +1}u^{\alpha (\beta -1)-1}dx}{(1-\alpha )\int_{0}^{\infty }x^{-2\alpha }{\left(1-u\right)}^{\alpha }u^{\alpha (\beta -1)}dx}-\frac{\alpha^{2}[\int_{0}^{\infty }x^{-2\alpha -1}{\left(1-u\right)}^{\alpha }u^{\alpha (\beta -1)-1}(\beta u-\beta +1)dx{]}^{2}}{(1-\alpha )[\int_{0}^{\infty }x^{-2\alpha }{\left(1-u\right)}^{\alpha }u^{\alpha (\beta -1)}dx{]}^{2}}\}, \end{array}$$

$$\begin{array}{l} \phi_{3\beta \lambda }=\phi_{3\lambda \beta }=2[\frac{\alpha }{\lambda (1-\alpha )}-\frac{\alpha \int_{0}^{\infty }x^{-2\alpha -1}{\left(1-u\right)}^{\alpha }u^{\alpha (\beta -1)-1}(\beta u-\beta +1)dx}{(1-\alpha )\int_{0}^{\infty }x^{-2\alpha }{\left(1-u\right)}^{\alpha }u^{\alpha (\beta -1)}dx}]\\ \;\times [\frac{\alpha }{\beta (1-\alpha )}+\frac{\alpha \int_{0}^{\infty }x^{-2\alpha }{\left(1-u\right)}^{\alpha }u^{\alpha (\beta -1)}\ln\;udx}{(1-\alpha )\int_{0}^{\infty }x^{-2\alpha }{\left(1-u\right)}^{\alpha }u^{\alpha (\beta -1)}dx}]\\ \;+2H_{R}(f)\{-\frac{\alpha^{2}\int_{0}^{\infty }x^{-2\alpha -1}{\left(1-u\right)}^{\alpha }u^{\alpha (\beta -1)-1}\ln\;u(\beta u-\beta +1)dx}{(1-\alpha )\int_{0}^{\infty }x^{-2\alpha }{\left(1-u\right)}^{\alpha }u^{\alpha (\beta -1)}dx}\\ \;+\frac{\alpha \int_{0}^{\infty }x^{-2\alpha -1}{\left(1-u\right)}^{\alpha +1}u^{\alpha (\beta -1)-1}dx}{(1-\alpha )\int_{0}^{\infty }x^{-2\alpha }{\left(1-u\right)}^{\alpha }u^{\alpha (\beta -1)}dx}\\ \;+\frac{\alpha^{2}\int_{0}^{\infty }x^{-2\alpha }{\left(1-u\right)}^{\alpha }u^{\alpha (\beta -1)}\ln\;udx\int_{0}^{\infty }x^{-2\alpha -1}{\left(1-u\right)}^{\alpha }u^{\alpha (\beta -1)-1}(\beta u-\beta +1)dx}{(1-\alpha ){\left[\int_{0}^{\infty }x^{-2\alpha }{\left(1-u\right)}^{\alpha }u^{\alpha (\beta -1)}dx\right]}^{2}}\}. \end{array}$$

Substitute the above equations into Eq (20) to obtain $E[H_{R}^{2}|\underline{x}]$. Next, we calculate *ϕ*_4_ = *H*_*R*_.


$$\phi_{4\beta }=\frac{\alpha }{\beta (1-\alpha )}+\frac{\alpha \int_{0}^{\infty }x^{-2\alpha }{\left(1-u\right)}^{\alpha }u^{\alpha (\beta -1)}\ln\;udx}{(1-\alpha )\int_{0}^{\infty }x^{-2\alpha }{\left(1-u\right)}^{\alpha }u^{\alpha (\beta -1)}dx},$$

$$\begin{array}{l} \phi_{4\beta \beta }=-\frac{\alpha }{\beta^{2}(1-\alpha )}+\frac{\alpha^{2}\int_{0}^{\infty }x^{-2\alpha }{\left(1-u\right)}^{\alpha }u^{\alpha (\beta -1)}{\left(\ln\;u\right)}^{2}dx}{(1-\alpha )\int_{0}^{\infty }x^{-2\alpha }{\left(1-u\right)}^{\alpha }u^{\alpha (\beta -1)}dx}\\ \;-\frac{\alpha^{2}{\left[\int_{0}^{\infty }x^{-2\alpha }{\left(1-u\right)}^{\alpha }u^{\alpha (\beta -1)}\ln\;udx\right]}^{2}}{(1-\alpha ){\left[\int_{0}^{\infty }x^{-2\alpha }{\left(1-u\right)}^{\alpha }u^{\alpha (\beta -1)}dx\right]}^{2}}, \end{array}$$

$$\phi_{4\lambda }=\frac{\alpha }{\lambda (1-\alpha )}-\frac{\alpha \int_{0}^{\infty }x^{-2\alpha -1}{\left(1-u\right)}^{\alpha }u^{\alpha (\beta -1)-1}(\beta u-\beta +1)dx}{(1-\alpha )\int_{0}^{\infty }x^{-2\alpha }{\left(1-u\right)}^{\alpha }u^{\alpha (\beta -1)}dx},$$

$$\begin{array}{l} \phi_{4\lambda \lambda }=-\frac{\alpha }{\lambda^{2}(1-\alpha )}+\frac{\alpha \int_{0}^{\infty }x^{-2\alpha -2}{\left(1-u\right)}^{\alpha }u^{\alpha (\beta -1)-2}(\beta u-\beta +1)(\alpha \beta u-\alpha \beta +\alpha -u+1)dx}{(1-\alpha )\int_{0}^{\infty }x^{-2\alpha }{\left(1-u\right)}^{\alpha }u^{\alpha (\beta -1)}dx}\\ \;-\frac{\alpha \beta \int_{0}^{\infty }x^{-2\alpha -2}{\left(1-u\right)}^{\alpha +1}u^{\alpha (\beta -1)-1}dx}{(1-\alpha )\int_{0}^{\infty }x^{-2\alpha }{\left(1-u\right)}^{\alpha }u^{\alpha (\beta -1)}dx}-\frac{\alpha^{2}{\left[\int_{0}^{\infty }x^{-2\alpha -1}{\left(1-u\right)}^{\alpha }u^{\alpha (\beta -1)-1}(\beta u-\beta +1)dx\right]}^{2}}{(1-\alpha ){\left[\int_{0}^{\infty }x^{-2\alpha }{\left(1-u\right)}^{\alpha }u^{\alpha (\beta -1)}dx\right]}^{2}}, \end{array}$$

$$\begin{array}{l} \phi_{4\beta \lambda }=\phi_{4\lambda \beta }=-\frac{\alpha^{2}\int_{0}^{\infty }x^{-2\alpha -1}{\left(1-u\right)}^{\alpha }u^{\alpha (\beta -1)-1}\ln\;u(\beta u-\beta +1)dx}{(1-\alpha )\int_{0}^{\infty }x^{-2\alpha }{\left(1-u\right)}^{\alpha }u^{\alpha (\beta -1)}dx}\\ \;+\frac{\alpha \int_{0}^{\infty }x^{-2\alpha -1}{\left(1-u\right)}^{\alpha +1}u^{\alpha (\beta -1)-1}dx}{(1-\alpha )\int_{0}^{\infty }x^{-2\alpha }{\left(1-u\right)}^{\alpha }u^{\alpha (\beta -1)}dx}\\ \;+\frac{\alpha^{2}\int_{0}^{\infty }x^{-2\alpha }{\left(1-u\right)}^{\alpha }u^{\alpha (\beta -1)}\ln\;udx\int_{0}^{\infty }x^{-2\alpha -1}{\left(1-u\right)}^{\alpha }u^{\alpha (\beta -1)-1}(\beta u-\beta +1)dx}{(1-\alpha ){\left[\int_{0}^{\infty }x^{-2\alpha }{\left(1-u\right)}^{\alpha }u^{\alpha (\beta -1)}dx\right]}^{2}}. \end{array}$$

Substitute the above equations into Eq (20) to obtain *E*[*H*_*R*_|*x*], and then the BE of Rényi entropy under DLF can be obtained from Eq (19).

## 5. Monte Carlo modeling

Monte Carlo simulation is a statistical simulation method based on random sampling, which generates a large number of random samples and simulates and infers based on these samples to obtain corresponding approximate results. In this section, we use the Monte Carlo method combined with the estimation method used in this paper to calculate the average estimates (AEs) and corresponding mean square errors (MSEs) of Shannon entropy and Rényi entropy. For further analysis, we used the bootstrap method to obtain the average width (AW) and coverage probability (CP) of the entropy CI. Firstly, we set the true values of the parameters *β* = 0.5, *λ* = 0.5, and the censoring schemes (see Table 3). Based on the given censoring schemes, we generate PC-II data using an algorithm (see Wang and Gui [40]) to calculate the AEs and MSEs of the GIED model parameters (see Tables 4 and 5). On this basis, we assume hyperparameters *a*_1_ = *b*_1_ = *a*_2_ = *b*_2_ = 1, entropy parameters c = 2, and *α* = 1.5 and conduct 1000 repeated experiments with different sample sizes to obtain ML estimates and Bayesian estimates of Shannon entropy and Rényi entropy, as well as corresponding MSEs (see Tables 6 and 7). For simplicity, we denote the ML estimates as MLE, and the Bayesian estimates under the three loss functions as LBe, EBe, and DBe. When constructing CIs using the bootstrap method, we set confidence levels *θ* = 0.05,0.1, and sampled the original data with replacement. The number of samples was set to *m*_1_ = 30,*m*_2_ = 50,*m*_3_ = 80, and the number of repeated samples was B = 5000. Tables 8 and 9 represent the AW and CP of Shannon entropy and Rényi entropy at 100(1−*θ*)% CIs, respectively. Through these analyses, we can gain a deeper understanding of the accuracy and reliability of entropy estimation.

**Table 3:** PC-II schemes.

Censoring Samples	
**Scheme a**	if *m* is odd: $R_{i}=0,R_{(m+1)/2}=n-m(i\neq (m+1)/2)$
	if *m* is even: $R_{i}=0,R_{m/2}=n-m(i\neq m/2)$
**Scheme b**	if *n* is odd and *m* is even, or if *n* is even and *m* is odd:
	$R_{1}=(n-m-1)/2,R_{2}=R_{3}=\cdots =R_{m-1}=0,R_{m}=(n-m-1)/2$
	if *n* and *m* are both odd (even):
	$R_{1}=(n-m)/2,R_{2}=R_{3}=\cdots =R_{m-1}=0,R_{m}=(n-m)/2$
**Scheme c**	$R_{1}=R_{2}=\cdots =R_{m}=1$

**Table 4:** The AEs and corresponding MSEs of *β* in the GIED model under different censoring schemes.

n	m	CS	MLE		Lindley					
					LBe		EBe		DBe	
			AE	MSE	AE	MSE	AE	MSE	AE	MSE
**30**	**15**	**a**	0.6209	0.0974	0.5858	0.0471	0.5657	0.0474	0.7073	0.1229
		**b**	0.6110	0.0872	0.5849	0.0452	0.5648	0.0452	0.7038	0.1209
		**c**	0.6031	0.0763	0.5757	0.0389	0.5549	0.0400	0.6985	0.1162
	**20**	**a**	0.5637	0.0396	0.5511	0.0267	0.5352	0.0268	0.6243	0.0554
		**b**	0.5811	0.0483	0.5677	0.0323	0.5471	0.0273	0.6370	0.0579
		**c**	0.5735	0.0539	0.5598	0.0328	0.5423	0.0324	0.6429	0.0707
	**25**	**a**	0.5475	0.0242	0.5411	0.0191	0.5282	0.0187	0.5932	0.0334
		**b**	0.5476	0.0255	0.5414	0.0197	0.5289	0.0195	0.5923	0.0343
		**c**	0.5616	0.0332	0.5545	0.0250	0.5399	0.0246	0.6192	0.0490
**50**	**20**	**a**	0.5916	0.0598	0.5720	0.0373	0.5556	0.0382	0.6618	0.0844
		**b**	0.5762	0.0510	0.5665	0.0330	0.5487	0.0335	0.6567	0.0811
		**c**	0.5791	0.0558	0.5640	0.0346	0.5471	0.0349	0.6502	0.0783
	**25**	**a**	0.5603	0.0307	0.5521	0.0228	0.5376	0.0224	0.6145	0.0439
		**b**	0.5536	0.0313	0.5490	0.0233	0.5399	0.0265	0.6127	0.0448
		**c**	0.5605	0.0348	0.5530	0.0253	0.5384	0.0252	0.6174	0.0497
	**40**	**a**	0.5337	0.0122	0.5316	0.0108	0.5229	0.0104	0.5627	0.0160
		**b**	0.5247	0.0109	0.5236	0.0097	0.5150	0.0094	0.5533	0.0140
		**c**	0.5398	0.0171	0.5383	0.0148	0.5283	0.0144	0.5754	0.0230
**80**	**25**	**a**	0.5631	0.0311	0.5539	0.0232	0.5381	0.0226	0.6232	0.0471
		**b**	0.5672	0.0445	0.5630	0.0308	0.5466	0.0308	0.6398	0.0683
		**c**	0.5659	0.0393	0.5575	0.0273	0.5430	0.0272	0.6231	0.0542
	**40**	**a**	0.5306	0.0159	0.5284	0.0137	0.5187	0.0134	0.5636	0.0205
		**b**	0.5357	0.0156	0.5358	0.0138	0.5261	0.0134	0.5712	0.0213
		**c**	0.5262	0.0139	0.5256	0.0123	0.5156	0.0119	0.5610	0.0186
	**60**	**a**	0.5177	0.0079	0.5172	0.0074	0.5111	0.0072	0.5373	0.0095
		**b**	0.5195	0.0082	0.5193	0.0077	0.5134	0.0075	0.5391	0.0099
		**c**	0.5231	0.0100	0.5231	0.0093	0.5162	0.0090	0.5464	0.0124

**Table 5:** The AEs and corresponding MSEs of *λ* in the GIED model under different censoring schemes.

n	m	CS	MLE		Lindley					
					LBe		EBe		DBe	
			AE	MSE	AE	MSE	AE	MSE	AE	MSE
**30**	**15**	**a**	0.5892	0.0546	0.5612	0.0378	0.5382	0.0392	0.6917	0.1050
		**b**	0.5823	0.0518	0.5561	0.0351	0.5307	0.0371	0.6796	0.1707
		**c**	0.5805	0.0501	0.5531	0.0326	0.5278	0.0347	0.6929	0.1026
	**20**	**a**	0.5743	0.0467	0.5617	0.0367	0.5440	0.0380	0.6383	0.0608
		**b**	0.5810	0.0483	0.5619	0.0332	0.5433	0.0340	0.6403	0.0566
		**c**	0.5646	0.0385	0.5508	0.0290	0.5310	0.0296	0.6360	0.0783
	**25**	**a**	0.5646	0.0377	0.5582	0.0332	0.5439	0.0340	0.6121	0.0452
		**b**	0.5651	0.0368	0.5589	0.0319	0.5453	0.0325	0.6113	0.0445
		**c**	0.5452	0.0233	0.5380	0.0188	0.5205	0.0190	0.6069	0.0355
**50**	**20**	**a**	0.5605	0.0321	0.5409	0.0212	0.5202	0.0220	0.6393	0.0535
		**b**	0.5593	0.0332	0.5496	0.0239	0.5276	0.0246	0.6469	0.0595
		**c**	0.5601	0.0346	0.5450	0.0235	0.5244	0.0244	0.6385	0.0558
	**25**	**a**	0.5496	0.0213	0.5414	0.0172	0.5247	0.0172	0.6072	0.0323
		**b**	0.5565	0.0309	0.5414	0.0223	0.5245	0.0226	0.6074	0.0391
		**c**	0.5510	0.0266	0.5435	0.0209	0.5265	0.0212	0.6114	0.0390
	**40**	**a**	0.5428	0.0184	0.5408	0.0171	0.5316	0.0171	0.5724	0.0216
		**b**	0.5370	0.0200	0.5359	0.0188	0.5269	0.0190	0.5660	0.0227
		**c**	0.5250	0.0128	0.5234	0.0115	0.5121	0.0114	0.5622	0.0168
**80**	**25**	**a**	0.5344	0.0159	0.5252	0.0159	0.5058	0.0123	0.5994	0.0272
		**b**	0.5457	0.0233	0.5415	0.0171	0.5212	0.0171	0.6245	0.0439
		**c**	0.5527	0.0261	0.5443	0.0206	0.5273	0.0208	0.6149	0.0404
	**40**	**a**	0.5268	0.0132	0.5246	0.0118	0.5141	0.0117	0.5607	0.0168
		**b**	0.5331	0.0151	0.5331	0.0139	0.5225	0.0138	0.5696	0.0196
		**c**	0.5229	0.0121	0.5223	0.0110	0.5113	0.0110	0.5587	0.0156
	**60**	**a**	0.5260	0.0110	0.5255	0.0105	0.5193	0.0105	0.5458	0.0124
		**b**	0.5254	0.0117	0.5253	0.0112	0.5191	0.0112	0.5453	0.0131
		**c**	0.5188	0.0088	0.5188	0.0083	0.5115	0.0082	0.5426	0.0106

**Table 6:** The AEs and corresponding MSEs of Shannon entropy under different censoring schemes.

n	m	CS	MLE		Lindley					
					LBe		EBe		DBe	
			AE	MSE	AE	MSE	AE	MSE	AE	MSE
**30**	**15**	**a**	2.9283	0.6138	2.6109	0.6308	2.8305	0.5634	3.1959	0.5475
		**b**	2.9442	0.6292	2.6219	0.6478	2.8363	0.5782	3.1940	0.5670
		**c**	2.9043	0.5971	2.5832	0.6479	2.8017	0.5539	3.1697	0.5185
	**20**	**a**	2.9691	0.4353	2.7140	0.4555	2.8962	0.4102	3.1586	0.4043
		**b**	2.9947	0.4096	2.7323	0.4319	2.9145	0.3905	3.1779	0.3900
		**c**	2.9683	0.4561	2.6915	0.4845	2.8822	0.4297	3.1688	0.4192
	**25**	**a**	3.0144	0.3269	2.7966	0.3400	2.9529	0.3148	3.1607	0.3166
		**b**	3.0026	0.3389	2.7876	0.3556	2.9419	0.3297	3.1474	0.3260
		**c**	3.0004	0.3667	2.7605	0.3813	2.9283	0.3461	3.1616	0.3451
**50**	**20**	**a**	2.9552	0.5191	2.6752	0.5345	2.8719	0.4786	3.1700	0.4713
		**b**	2.9973	0.4849	2.7023	0.5045	2.8936	0.4519	3.1908	0.4453
		**c**	2.9688	0.4767	2.6921	0.4976	2.8821	0.4448	3.1681	0.4362
	**25**	**a**	2.9876	0.3669	2.7516	0.3878	2.9197	0.3493	3.1525	0.3431
		**b**	2.9852	0.3841	2.7535	0.4038	2.9142	0.3699	3.1373	0.3591
		**c**	3.0122	0.3792	2.7693	0.3900	2.9378	0.3568	3.1724	0.3578
	**40**	**a**	3.0351	0.1892	2.8818	0.1981	2.9946	0.1851	3.1291	0.1856
		**b**	3.0330	0.2211	2.8802	0.2267	2.9917	0.2159	3.1249	0.2154
		**c**	3.0127	0.2458	2.8453	0.2567	2.9661	0.2392	3.1152	0.2338
**80**	**25**	**a**	2.9827	0.4112	2.7225	0.4335	2.9056	0.3867	3.1698	0.3834
		**b**	2.9799	0.3931	2.7052	0.4381	2.8792	0.3819	3.1439	0.3553
		**c**	2.9777	0.3680	2.7398	0.3963	2.9060	0.3535	3.1394	0.3394
	**40**	**a**	3.0138	0.2439	2.8481	0.2530	2.9696	0.2366	3.1187	0.2334
		**b**	3.0233	0.2475	2.8568	0.2557	2.9741	0.2414	3.1187	0.2375
		**c**	3.0268	0.2135	2.8566	0.2251	2.9789	0.2074	3.1295	0.2060
	**60**	**a**	3.0531	0.1506	2.9423	0.1519	3.0248	0.1478	3.1169	0.1487
		**b**	3.0535	0.1515	2.9441	0.1530	3.0252	0.1491	3.1156	0.1498
		**c**	3.0159	0.1554	2.8966	0.1661	2.9843	0.1542	3.0852	0.1482

**Table 7:** The AEs and corresponding MSEs of Rényi entropy under different censoring schemes.

n	m	CS	MLE		Lindley					
					LBe		EBe		DBe	
			AE	MSE	AE	MSE	AE	MSE	AE	MSE
**30**	**15**	**a**	1.8957	0.2241	1.7651	0.2261	1.8313	0.2251	2.0255	0.1884
		**b**	1.9177	0.2122	1.7828	0.2156	1.8493	0.2167	2.0421	0.1852
		**c**	1.8999	0.2217	1.7692	0.2241	1.8352	0.2230	2.0281	0.1868
	**20**	**a**	1.9556	0.1667	1.8435	0.1669	1.8995	0.1686	2.0457	0.1550
		**b**	1.9472	0.1578	1.8353	0.1619	1.8912	0.1622	2.0381	0.1465
		**c**	1.9217	0.1539	1.8124	0.1610	1.8672	0.1580	2.0146	0.1351
	**25**	**a**	1.9654	0.1292	1.8675	0.1314	1.9160	0.1317	2.0367	0.1232
		**b**	1.9763	0.1294	1.8776	0.1301	1.9268	0.1310	2.0483	0.1245
		**c**	1.9450	0.1230	1.8533	0.1270	1.9001	0.1253	2.0177	0.1125
**50**	**20**	**a**	1.9209	0.1612	1.8114	0.1659	1.8678	0.1634	2.0200	0.1402
		**b**	1.9157	0.1640	1.8030	0.1742	1.8577	0.1707	2.0075	0.1439
		**c**	1.9227	0.1651	1.8133	0.1709	1.8680	0.1690	2.0155	0.1460
	**25**	**a**	1.9521	0.1123	1.8599	0.1167	1.9073	0.1148	2.0255	0.1037
		**b**	1.9456	0.1244	1.8530	0.1296	1.8996	0.1282	2.0170	0.1153
		**c**	1.9395	0.1182	1.8468	0.1243	1.8935	0.1216	2.0119	0.1075
	**40**	**a**	1.9797	0.0780	1.9158	0.0786	1.9486	0.0788	2.0235	0.0759
		**b**	1.9818	0.0859	1.9170	0.0863	1.9502	0.0869	2.0266	0.0838
		**c**	1.9666	0.0771	1.9050	0.0786	1.9370	0.0780	2.0109	0.0734
**80**	**25**	**a**	1.9385	0.1347	1.8429	0.1365	1.8930	0.1346	2.0214	0.1196
		**b**	1.9322	0.1378	1.8300	0.1456	1.8777	0.1428	2.0042	0.1221
		**c**	1.9507	0.1258	1.8577	0.1297	1.9047	0.1286	2.0230	0.1162
	**40**	**a**	1.9719	0.0716	1.9111	0.0728	1.9433	0.0722	2.0171	0.0684
		**b**	1.9612	0.0783	1.8994	0.0808	1.9311	0.0800	2.0046	0.0745
		**c**	1.9509	0.0836	1.8894	0.0868	1.9210	0.0855	1.9954	0.0782
	**60**	**a**	1.9809	0.0482	1.9371	0.0492	1.9599	0.0489	2.0099	0.0472
		**b**	1.9859	0.0500	1.9415	0.0505	1.9647	0.0505	2.0155	0.0493
		**c**	1.9829	0.0493	1.9395	0.0498	1.9621	0.0497	2.0118	0.0482

**Table 8:** AW and CP of the 100(1−*θ*)% CI for Shannon entropy.

n	m	CS	*θ* = 0.05		*θ* = 0.1	
			AW	CP	AW	CP
**30**	**15**	**a**	5.3239	0.9920	6.6893	1.0000
		**b**	4.0999	0.8170	5.9816	0.9580
		**c**	3.6197	0.9490	3.8822	0.9710
	**20**	**a**	3.7924	0.9720	6.6814	1.0000
		**b**	3.0828	0.7500	3.8277	0.9850
		**c**	2.3251	0.8240	2.3413	0.9130
	**25**	**a**	2.0543	0.9000	2.6363	0.9740
		**b**	1.9814	0.8670	1.9961	0.8920
		**c**	1.9010	0.8210	1.9616	0.8570
**50**	**20**	**a**	5.4345	0.9980	6.3533	1.0000
		**b**	3.5983	0.9390	7.7951	0.9890
		**c**	2.2638	0.8750	2.5799	0.9170
	**25**	**a**	4.3206	0.9980	5.5457	1.0000
		**b**	3.5080	0.8680	5.2608	1.0000
		**c**	2.0171	0.7200	2.3511	0.8330
	**40**	**a**	2.2185	0.8810	2.3712	0.9830
		**b**	1.7983	0.9460	2.1869	0.9560
		**c**	1.7255	0.8270	2.3354	0.9200
**80**	**25**	**a**	8.3366	1.0000	8.7971	1.0000
		**b**	7.0129	0.9990	12.4071	1.0000
		**c**	2.0614	0.8410	2.2768	0.8790
	**40**	**a**	3.5499	1.0000	7.2944	1.0000
		**b**	2.1869	0.9560	3.0937	0.9820
		**c**	1.7966	0.8850	1.9492	0.9100
	**60**	**a**	2.9989	0.9970	4.0421	1.0000
		**b**	1.6839	0.9000	1.7348	0.9740
		**c**	1.2961	0.7980	1.3215	0.8560

**Table 9:** AW and CP of the 100(1−*θ*)% CI for Rényi entropy.

n	m	CS	*θ* = 0.05		*θ* = 0.1	
			AW	CP	AW	CP
**30**	**15**	**a**	3.3166	0.7950	3.7192	0.9900
		**b**	2.4433	0.8080	2.8280	0.9980
		**c**	1.4330	0.8890	1.7525	0.9260
	**20**	**a**	2.2759	0.9790	2.2837	0.9960
		**b**	1.6112	0.9670	1.6928	0.9700
		**c**	1.1083	0.7960	1.2660	0.8620
	**25**	**a**	1.5252	0.8090	1.5432	0.9430
		**b**	1.2712	0.8190	1.5312	0.9010
		**c**	1.0760	0.8570	1.1813	0.9160
**50**	**20**	**a**	3.2180	0.9980	4.1871	1.0000
		**b**	2.2686	0.8020	3.0443	0.9380
		**c**	1.6407	0.8050	1.8054	0.9580
	**25**	**a**	2.9590	0.9990	2.9933	0.9990
		**b**	2.0828	0.9010	2.1256	0.9980
		**c**	1.0752	0.8230	1.3139	0.9350
	**40**	**a**	1.6615	0.9810	1.6950	0.9820
		**b**	1.1528	0.9220	1.1572	0.9680
		**c**	0.9687	0.8220	1.0331	0.9200
**80**	**25**	**a**	3.5634	1.0000	4.8036	1.0000
		**b**	2.6304	0.9480	2.6695	0.9840
		**c**	1.2011	0.8610	1.5777	0.8780
	**40**	**a**	3.0343	1.0000	3.0852	1.0000
		**b**	1.5593	0.8650	2.0543	0.9990
		**c**	0.9525	0.8590	1.1215	0.9690
	**60**	**a**	1.4652	0.9660	1.6882	0.9980
		**b**	1.1043	0.9750	1.1169	0.9810
		**c**	0.7368	0.8090	0.9283	0.8710

Drawing upon the data presented in the aforementioned tables, the following research findings can be deduced:

(1) In parameter estimation and entropy estimation, Bayesian estimation performs better than ML estimation on the whole. Specifically, for parameter estimation, Bayesian estimation under LLF has the best performance. For entropy estimation, the Bayesian estimation under DLF has the best performance.

(2) When the total sample observations were fixed, the MSE of parameter estimation and entropy estimation showed a downward trend with the increase of the observed sample size, indicating that the accuracy of estimation increased with the increase of sample size.

(3) Through the analysis of Tables 8 and 9, it can be observed that AW of the CI of Shannon entropy and Rényi entropy gradually decreases with the increase of the observed sample size, while AW increases correspondingly with the increase of the confidence level. At the same time, CP of Shannon entropy and Rényi entropy increases with the increase of confidence level, especially when *θ* = 0.1, CP reaches the highest value.

## 6. Analyzing the data

Here, the methods of estimation used with this paper are demonstrated using actual data. The data set is as follows: 1.05, 2.92, 3.61, 4.20, 4.49, 6.72, 7.31, 9.08, 9.11, 14.49, 16.85, 18.82, 26.59, 30.26, 41.34. These data represent the survival time (in months) of Hodgkin’s disease patients undergoing intensive treatment with nitrogen mustard. Please refer to Bakoban and Abubaker [41] for more details. In order to assess the suitability of the GIED model for this dataset, we computed the Kolmogorov-Smirnov (KS) statistic for GIED, inverse Weibull distribution (IWD), exponential Weibull distribution (EWD), and Weibull distribution (WD) based on this dataset. The P-value derived from the KS statistic was utilized as a criterion to determine the optimal model selection. The specific values are presented in Table 10. Subsequently, Fig 3 illustrates both the empirical distribution of the dataset and cumulative distribution functions corresponding to each distribution model. It is evident from Table 10 and Fig 3 that the GIED model provides a reasonable fit for this dataset.

**Table 10:** ML estimates and goodness of fit testing under real data.

Model	*β*	*λ*	KS	P
**GIED**	1.2225	6.0791	0.0878	0.7934
**IWD**	1.0070	5.3734	0.1190	0.6541
**EWD**	0.4413	9.0813	0.0888	0.7893
**WD**	0.4798	2.5122	0.5253	0.0003

**Fig 4:**
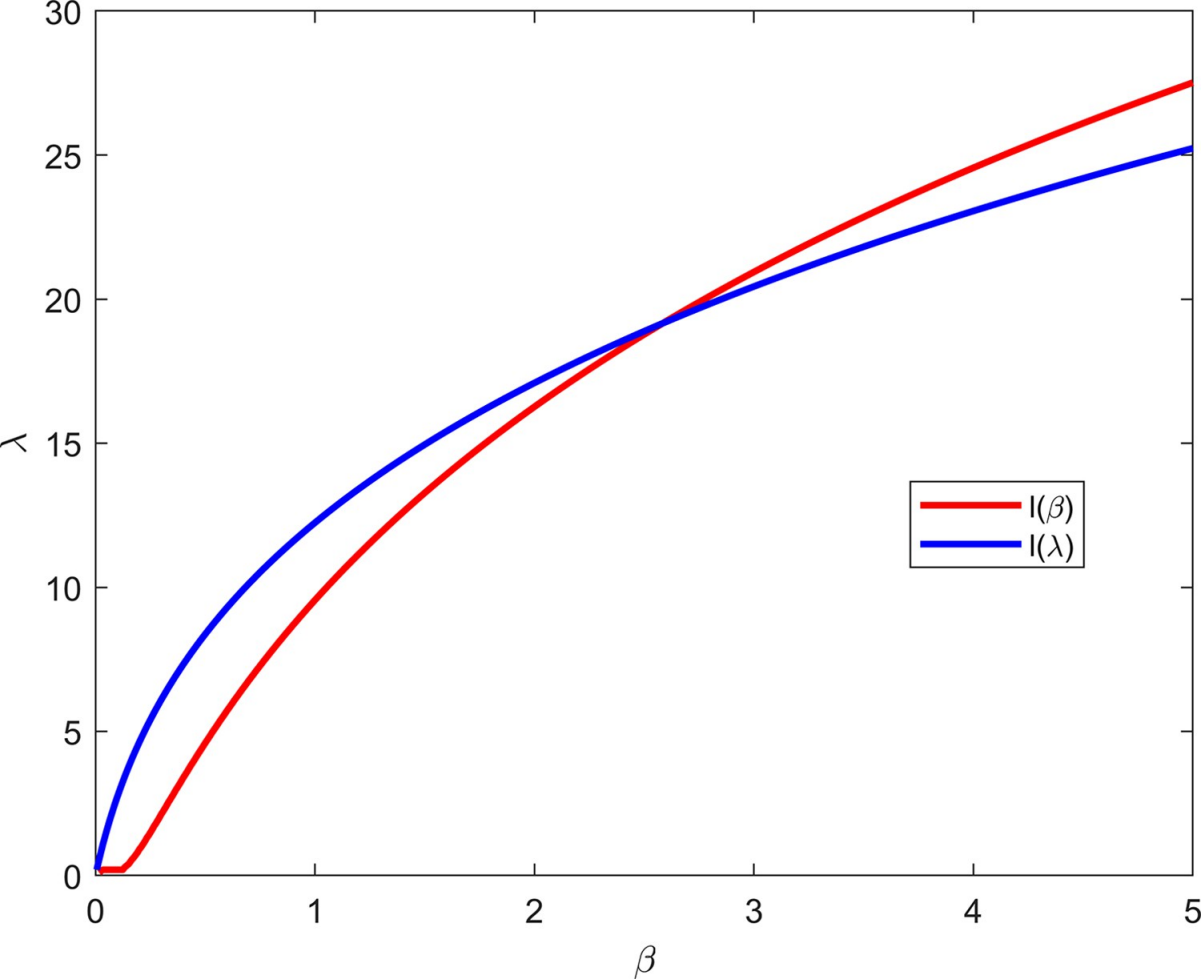
Partial derivatives of the log-LF.

To validate the performance of the proposed estimation method, and considering the validity of the data and the diversity of censoring schemes, we randomly selected m = 7 observations from the provided real dataset. The selection of m = 7 as the observation value is based on the optimization consideration of model performance. Through simulation experiments, we found that this value can maintain reasonable computational efficiency while ensuring prediction accuracy, and can better adapt to the characteristics and requirements of our dataset. Subsequently, according to the censoring schemes defined in Table 3, these observations were subjected to corresponding censoring processing to generate a more stable sample dataset, thereby providing a basis for subsequent statistical analysis, as detailed in Table 11. To demonstrate the solution of the ML estimation exists and is unique, we chose censoring scheme II and visualized the log-LF. Please refer to Fig 4 for details. Table 12 shows the ML estimates and the Bayesian estimates of the entropies on the basis of the actual data, with hyperparameters set as *a*_1_ = *b*_1_ = *a*_2_ = *b*_2_ = 1,*c* = 2, and *α* = 1.5. Table 13 presents the upper and lower bounds of the bootstrap CIs for entropy on the basis of the actual data at different confidence levels, with the number of repeated samples set to 5000.

**Table 11:** PC-II samples obtained using m = 7 observations.

Censoring Schemes	(*R*_1_,*R*_2_,*R*_3_,*R*_4_,*R*_5_,*R*_6_,*R*_7_)	Censoring samples
**Ⅰ**	(0,0,0,8,0,0,0)	1.05,2.92,3.61,4.20,26.59,30.26,41.34
**Ⅱ**	(4,0,0,0,0,0,4)	4.49,6.72,7.31,9.08,9.11,14.49,16.85
**Ⅲ**	(1,1,1,1,1,1,1)	2.92,4.20,6.72,9.08,14.49,18.82,30.26

**Table 12:** ML estimates and Bayesian estimates of Shannon entropy and Rényi entropy.

**Function**	**CS**	**MLE**	**Lindley**		
			**LBe**	**Ebe**	**Dbe**
**Shannon**	**Ⅰ**	4.5186	4.5166	5.4925	5.5425
	**Ⅱ**	3.7140	2.9786	16.8070	5.4097
	**Ⅲ**	4.4610	4.2071	7.5096	5.9143
**Rényi**	**Ⅰ**	3.7181	3.6707	3.9536	4.1057
	**Ⅱ**	3.4670	2.9455	8.5171	4.8137
	**Ⅲ**	3.9482	4.7925	4.9617	4.7266

**Table 13:** Upper and lower bounds of bootstrap CIs for Shannon entropy and Rényi entropy at different confidence levels.

Function	CS	*θ* = 0.05		*θ* = 0.1	
		Lower	Upper	Lower	Upper
**Shannon**	**Ⅰ**	1.2485	9.3561	1.8492	8.9169
	**Ⅱ**	-3.4156	7.4638	0.2626	5.3264
	**Ⅲ**	2.8578	5.5116	3.1250	5.2558
**Rényi**	**Ⅰ**	2.4808	5.4712	2.7526	5.1910
	**Ⅱ**	0.6082	4.3989	1.7767	3.8861
	**Ⅲ**	2.8755	4.6825	3.1935	4.4192

## 7. Conclusions

Within this paper, we discussed the estimation of entropy for the GIED’s Shannon entropy and Rényi entropy based on PC-II samples. We first introduced the PC-II experiment and the GIED model, and deduced the ML estimation expressions for entropy. Due to the invariance property of ML estimation, we used a dichotomy method to obtain the ML estimates of the parameters. Next, or the purpose of evaluating the accuracy and precision of the estimates of Shannon entropy and Rényi entropy, we used the bootstrap method to obtain the CIs of Shannon entropy and Rényi entropy. In Bayesian estimation, we introduced three loss functions, LLF, ELF, and DLF, to assess the disparities between the approximated values and actual values, helping us assess the performance of the model and make optimal choices. However, due to the complexity of the Bayesian estimation forms for entropy under the loss functions, direct computation was challenging. Therefore, we used the Lindley approximation algorithm to estimate their Bayesian estimates. Finally, we conducted simulation experiments using the Monte Carlo method to obtain the estimates and corresponding MSEs of Shannon entropy and Rényi entropy, and analyzed and compared the performance of different estimation methods used under the censoring schemes.

The analysis of GIED entropy estimation in PC-II samples can help us gain a deeper understanding and describe the uncertainty and information requirements of GIED in such sample scenarios. These measures provide an evaluation of the distribution characteristics, enabling a more comprehensive understanding of the information content and statistical features of censoring sample data in analysis and modeling. Furthermore, with the obtained entropy values, we can better comprehend and analyze the data characteristics in PC-II samples and make model selection, parameter estimation, and predictive analysis in relevant applications. This enhances our overall understanding and interpretability of the data, improving our ability to comprehend and interpret the data comprehensively. Furthermore, the estimation of entropy in distribution models plays a crucial role in evaluating product reliability. A low entropy value indicates a high level of stability in the product’s lifespan, thereby reflecting its superior reliability. Conversely, a high entropy value suggests the need for optimization and improvement of the product. Entropy is also applicable to assess the risk of product failure since higher entropy signifies increased uncertainty regarding the product’s lifespan and consequently elevates the risk of failure. Based on this understanding, targeted risk control strategies can be developed to minimize potential risks.

## Supporting information

S1 Appendix Prove Theorem 1. (DOCX)

S2 Appendix Prove Theorem 2. (DOCX)
