# Supplementary material for: Statistical inference of entropy functions of generalized inverse exponential model under progressive type-II censoring test
Source: PLoS One. 2024 Sep 30;19(9):e0311129. doi: 10.1371/journal.pone.0311129 (PMC11441688; doi:10.1371/journal.pone.0311129)
Supplement: S1 Appendix — (DOCX) [file pone.0311129.s001.docx]

**Appendix A**

It can be deduced from Equation (1):

$$\ln f(x)=\ln[\frac{\lambda\beta}{x^{2}}\mathrm{e}^{-\frac{\lambda}{x}}(1-\mathrm{e}^{-\frac{\lambda}{x}})^{\beta-1}]=\ln\lambda\beta-2\ln x-\frac{\lambda}{x}+(\beta-1)\ln(1-\mathrm{e}^{-\frac{\lambda}{x}}).$$

Substituting Equation (21) into Equation (3), we can obtain:

$$\begin{matrix} H_{S}=-\int_{0}^{\infty} f(x)[\ln\lambda\beta-2\ln x-\frac{\lambda}{x}+(\beta-1)\ln(1-\mathrm{e}^{-\frac{\lambda}{x}})]dx \\ \text{ }=-\int_{0}^{\infty} \ln\lambda\beta f(x)dx+2\int_{0}^{\infty} \ln xf(x)dx+\int_{0}^{\infty} \frac{\lambda}{x}f(x)dx-(\beta-1)\int_{0}^{\infty} \ln(1-\mathrm{e}^{-\frac{\lambda}{x}})f(x)dx \\ \text{ }=-\ln\lambda\beta+2E[\ln X]+E[\frac{\lambda}{X}]-(\beta-1)E[\ln(1-\mathrm{e}^{-\frac{\lambda}{X}})]. \end{matrix}$$

Clearly, $\int_{0}^{\infty} f(x)dx=1$ , i.e.,

$$\int_{0}^{\infty} \frac{\lambda\beta}{x^{2}}\mathrm{e}^{-\frac{\lambda}{x}}(1-\mathrm{e}^{-\frac{\lambda}{x}})^{\beta-1}dx=1.$$

Upon differentiating Equation (22) with respect to $\beta$ on both sides, we have:

$$\int_{0}^{\infty} [\frac{\lambda}{x^{2}}\mathrm{e}^{-\frac{\lambda}{x}}(1-\mathrm{e}^{-\frac{\lambda}{x}})^{\beta-1}+\frac{\lambda\beta}{x^{2}}\mathrm{e}^{-\frac{\lambda}{x}}(1-\mathrm{e}^{-\frac{\lambda}{x}})^{\beta-1}\ln(1-\mathrm{e}^{-\frac{\lambda}{x}})]dx=0.$$

Thus,

$$\int_{0}^{\infty} \frac{\lambda\beta}{x^{2}}\mathrm{e}^{-\frac{\lambda}{x}}(1-\mathrm{e}^{-\frac{\lambda}{x}})^{\beta-1}\ln(1-\mathrm{e}^{-\frac{\lambda}{x}})dx=-\frac{1}{\beta}.$$

Then, we have:

$$E[\ln(1-\mathrm{e}^{-\frac{\lambda}{X}})]=-\frac{1}{\beta}.$$

Due to

$$E[\frac{\lambda}{X}]=\int_{0}^{\infty} \frac{\lambda}{x}[\frac{\lambda\beta}{x^{2}}\mathrm{e}^{-\frac{\lambda}{x}}(1-\mathrm{e}^{-\frac{\lambda}{x}})^{\beta-1}]dx=-\int_{0}^{\infty} \beta\frac{\lambda}{x}\mathrm{e}^{-\frac{\lambda}{x}}(1-\mathrm{e}^{-\frac{\lambda}{x}})^{\beta-1}d(\frac{\lambda}{x}).$$

Letting $\omega=\frac{\lambda}{X}$ , we have:

$$E[\omega]=-\int_{\infty}^{0} \beta\omega\mathrm{e}^{-\omega}(1-\mathrm{e}^{-\omega})^{\beta-1}d\omega=\int_{0}^{\infty} \beta\omega\mathrm{e}^{-\omega}(1-\mathrm{e}^{-\omega})^{\beta-1}d\omega.$$

The PDF of the GED is given by:

Thus

Letting $\nu=\frac{t}{\sigma}$ , we have:

From this, we can determine $E[\omega]=\psi(\beta+1)-\psi(1)$ .

Next, we calculate

$$E[X^{r}]=\int_{0}^{\infty} x^{r}[\frac{\lambda\beta}{x^{2}}\mathrm{e}^{-\frac{\lambda}{x}}(1-\mathrm{e}^{-\frac{\lambda}{x}})^{\beta-1}]dx=-\int_{0}^{\infty} x^{r}\beta\mathrm{e}^{-\frac{\lambda}{x}}(1-\mathrm{e}^{-\frac{\lambda}{x}})^{\beta-1}d(\frac{\lambda}{x}).$$

Letting $t=\frac{\lambda}{x}$ , we have $x=\frac{\lambda}{t}$ .

Thus

$$\begin{matrix} -\int_{0}^{\infty} x^{r}\beta\mathrm{e}^{-\frac{\lambda}{x}}(1-\mathrm{e}^{-\frac{\lambda}{x}})^{\beta-1}d(\frac{\lambda}{x})=-\int_{\infty}^{0} (\frac{\lambda}{t})^{r}\beta\mathrm{e}^{-t}(1-\mathrm{e}^{-t})^{\beta-1}dt \\ \text{ }=\lambda^{r}\beta\int_{0}^{\infty} t^{-r}\mathrm{e}^{-t}(1-\mathrm{e}^{-t})^{\beta-1}dt. \end{matrix}$$

Continuing, let $u=\mathrm{e}^{-t}$ , then $t=-\ln u,dt=-\frac{1}{u}du$ .

Thus

$$\begin{matrix} \lambda^{r}\beta\int_{0}^{\infty} t^{-r}e^{-t}(1-e^{-t})^{\beta-1}dt=\lambda^{r}\beta\int_{1}^{0} (-\ln u)^{-r}u(1-u)^{\beta-1}(-\frac{1}{u})du \\ \text{ }=\lambda^{r}\beta\int_{0}^{1} (-\ln u)^{-r}(1-u)^{\beta-1}du. \end{matrix}$$

Decomposition of $(1-u)^{\beta-1}$ into polynomials:

$$(1-u)^{\beta-1}=\sum_{i=0}^{\infty} \left(\begin{matrix} \beta-1 \\ i \end{matrix}\right)(-1)^{i}u^{i}.$$

Thus

$$\begin{matrix} \lambda^{r}\beta\int_{0}^{1} (-\ln u)^{-r}(1-u)^{\beta-1}du=\lambda^{r}\beta\int_{0}^{1} (-\ln u)^{-r}\sum_{i=0}^{\infty} \left(\begin{matrix} \beta-1 \\ i \end{matrix}\right)(-1)^{i}u^{i}du \\ \text{ }=\lambda^{r}\beta\sum_{i=0}^{\infty} \left(\begin{matrix} \beta-1 \\ i \end{matrix}\right)(-1)^{i}\int_{0}^{1} (-\ln u)^{-r}u^{i}du. \end{matrix}$$

From the negative logarithm gamma distribution, we have:

$$\int_{0}^{1} (-\ln u)^{-r}u^{i}du=\frac{\Gamma(1-r)}{(1+i)^{1-r}}.$$

Thus

$$\lambda^{r}\beta\sum_{i=0}^{\infty} \left(\begin{matrix} \beta-1 \\ i \end{matrix}\right)(-1)^{i}\int_{0}^{1} (-\ln u)^{-r}u^{i}du=\lambda^{r}\beta\sum_{i=0}^{\infty} \left(\begin{matrix} \beta-1 \\ i \end{matrix}\right)(-1)^{i}\frac{\Gamma(1-r)}{(1+i)^{1-r}}.$$

$$E[X^{r}]=\lambda^{r}\beta\sum_{i=0}^{\infty} \left(\begin{matrix} \beta-1 \\ i \end{matrix}\right)(-1)^{i}\frac{\Gamma(1-r)}{(1+i)^{1-r}},$$

$$\begin{matrix} \frac{dE[X^{r}]}{dr}=E[X^{r}\ln X]=(\lambda^{r}\ln\lambda)\beta\sum_{i=0}^{\infty} \left(\begin{matrix} \beta-1 \\ i \end{matrix}\right)(-1)^{i}\frac{\Gamma(1-r)}{(1+i)^{1-r}} \\ \text{ }+\lambda^{r}\beta\sum_{i=0}^{\infty} \left(\begin{matrix} \beta-1 \\ i \end{matrix}\right)(-1)^{i}\frac{-\Gamma'(1-r)(1+i)^{1-r}+\Gamma(1-r)(1+i)^{1-r}\ln(1+i)}{(1+i)^{2-2r}}. \end{matrix}$$

If r = 0, then

$$E[\ln X]=\beta\sum_{i=0}^{\infty} \left(\begin{matrix} \beta-1 \\ i \end{matrix}\right)(-1)^{i}\frac{\Gamma(1)\ln(1+i)-\Gamma'(1)}{1+i}.$$

As a result, we obtain:

$$\begin{matrix} H_{S}=-\ln\lambda\beta+2E[\ln X]+E[\frac{\lambda}{X}]-(\beta-1)E[\ln(1-e^{-\frac{\lambda}{X}})] \\ \text{ }=-\ln\lambda\beta+2\beta\sum_{i=0}^{\infty} \left(\begin{matrix} \beta-1 \\ i \end{matrix}\right)(-1)^{i}\frac{\Gamma(1)\ln(1+i)-\Gamma'(1)}{1+i}+\psi(\beta+1)-\psi(1)+\frac{\beta-1}{\beta}. \end{matrix}$$

Theorem 1 is proven. □
