# Supplementary material for: Statistical inference of entropy functions of generalized inverse exponential model under progressive type-II censoring test
Source: PLoS One. 2024 Sep 30;19(9):e0311129. doi: 10.1371/journal.pone.0311129 (PMC11441688; doi:10.1371/journal.pone.0311129)
Supplement: S2 Appendix — (DOCX) [file pone.0311129.s002.docx]

**Appendix B**

Substituting Equation (1) into Equation (4), we have:

$$\int_{0}^{\infty} [f(x)]^{\alpha}dx=\int_{0}^{\infty} [\frac{\lambda\beta}{x^{2}}\mathrm{e}^{-\frac{\lambda}{x}}(1-\mathrm{e}^{-\frac{\lambda}{x}})^{\beta-1}]^{\alpha}dx=(\lambda\beta)^{\alpha}\int_{0}^{\infty} x^{-2\alpha}\mathrm{e}^{-\frac{\lambda\alpha}{x}}(1-\mathrm{e}^{-\frac{\lambda}{x}})^{\alpha(\beta-1)}dx.$$

Letting $z=e^{-\frac{\lambda}{x}}$, we have $x=-\frac{\lambda}{\ln z},dx=\lambda z^{-1}(\ln z)^{-2}dz$.

Thus

$$\begin{matrix} (\lambda\beta)^{\alpha}\int_{0}^{\infty} x^{-2\alpha}e^{-\frac{\lambda\alpha}{x}}(1-e^{-\frac{\lambda}{x}})^{\alpha(\beta-1)}dx=(\lambda\beta)^{\alpha}\int_{1}^{0} (-\frac{\lambda}{\ln z})^{-2\alpha}z^{\alpha}(1-z)^{\alpha(\beta-1)}\lambda z^{-1}(-\ln z)^{-2}dz \\ \text{ }=\lambda^{-\alpha+1}\beta^{\alpha}\int_{1}^{0} (-\ln z)^{2\alpha-2}z^{\alpha-1}(1-z)^{\alpha(\beta-1)}dz. \end{matrix}$$

Decomposition of $(1-z)^{\alpha(\beta-1)}$ into polynomials:

$$(1-z)^{\alpha(\beta-1)}=\sum_{i=0}^{\infty} \left(\begin{matrix} \alpha\beta-\alpha\\ i \end{matrix}\right)(-1)^{i}z^{i}.$$

Thus

$$\begin{matrix} \lambda^{-\alpha+1}\beta^{\alpha}\int_{1}^{0} (-\ln z)^{2\alpha-2}z^{\alpha-1}(1-z)^{\alpha(\beta-1)}dz=\lambda^{-\alpha+1}\beta^{\alpha}\int_{1}^{0} (-\ln z)^{2\alpha-2}z^{\alpha-1}\sum_{i=0}^{\infty} \left(\begin{matrix} \alpha\beta-\alpha\\ i \end{matrix}\right)(-1)^{i}z^{i}dz \\ \text{ }=\lambda^{-\alpha+1}\beta^{\alpha}\sum_{i=0}^{\infty} \left(\begin{matrix} \alpha\beta-\alpha\\ i \end{matrix}\right)(-1)^{i}\int_{1}^{0} (-\ln z)^{2\alpha-2}z^{\alpha+i-1}dz \\ \text{ }=-\lambda^{-\alpha+1}\beta^{\alpha}\sum_{i=0}^{\infty} \left(\begin{matrix} \alpha\beta-\alpha\\ i \end{matrix}\right)(-1)^{i}\int_{0}^{1} (-\ln z)^{2\alpha-2}z^{\alpha+i-1}dz. \end{matrix}$$

From the negative logarithm gamma distribution, we have:

$$\int_{0}^{1} (-\ln z)^{2\alpha-2}z^{\alpha+i-1}dz=\frac{\Gamma(2\alpha-1)}{(\alpha+i)^{2\alpha-1}}.$$

Thus

$$-\lambda^{-\alpha+1}\beta^{\alpha}\sum_{i=0}^{\infty} \left(\begin{matrix} \alpha\beta-\alpha\\ i \end{matrix}\right)(-1)^{i}\int_{0}^{1} (-\ln z)^{2\alpha-2}z^{\alpha+i-1}dz=-\lambda^{-\alpha+1}\beta^{\alpha}\sum_{i=0}^{\infty} \left(\begin{matrix} \alpha\beta-\alpha\\ i \end{matrix}\right)(-1)^{i}\frac{\Gamma(2\alpha-1)}{(\alpha+i)^{2\alpha-1}}$$

As a result, we obtain:

$$H_{R}=\frac{1}{1-\alpha}\ln[-\lambda^{-\alpha+1}\beta^{\alpha}\sum_{i=0}^{\infty} \left(\begin{matrix} \alpha\beta-\alpha\\ i \end{matrix}\right)(-1)^{i}\frac{\Gamma(2\alpha-1)}{(\alpha+i)^{2\alpha-1}}].$$

Theorem 2 is proven. □
